# EVI1 promotes cell proliferation in HBx-induced hepatocarcinogenesis as a critical transcription factor regulating lncRNAs

**DOI:** 10.18632/oncotarget.7993

**Published:** 2016-03-08

**Authors:** Jin-feng Huang, Yue Wang, Feng Liu, Yin Liu, Chen-xi Zhao, Ying-jun Guo, Shu-han Sun

**Affiliations:** ^1^ The Department of Medical Genetics, Second Military Medical University, Shanghai, China

**Keywords:** ecotropic viral integration site 1, hepatocellular carcinoma, hepatitis B virus X protein, long non-coding RNA, transcription factor

## Abstract

The involvement of the hepatitis B virus X (HBx) protein in epigenetic modifications during hepatocarcinogenesis has been previously characterized. Long noncoding RNAs (lncRNAs), a kind of epigenetic regulator molecules, have also been shown to play crucial roles in HBx-related hepatocellular carcinoma (HCC). In this study, we analyzed the key transcription factors of aberrantly expressed lncRNAs in the livers of HBx transgenic mice by bioinformatics prediction, and found that ecotropic viral integration site 1 (Evi1) was a potential main transcription regulator. Further investigation showed that EVI1 was positively correlated to HBx expression and was frequently up-regulated in HBV-related HCC tissues. The forced expression of HBx in liver cell lines resulted in a significant increase of the expression of EVI1. Furthermore, suppression of EVI1 expression decreased the proliferation of HCC cells overexpressing HBx *in vitro* and *in vivo*.

**Conclusion:** Our findings suggest that EVI1 is frequently up-regulated and regulates a cluster of lncRNAs in HBV-related hepatocellular carcinoma (HCC). These findings highlight a novel mechanism for HBx-induced hepatocarcinogenesis through transcription factor EVI1 and its target lncRNAs, and provide a potential new approach to predict the functions of lncRNAs.

## INTRODUCTION

Hepatocellular carcinoma (HCC) is a prevalent cancer in the world, especially in Asia and Africa. Chronic hepatitis B virus (HBV) infection is responsible for the great majority of HCC in these areas, which can result in end-stage liver disease, including liver cirrhosis and HCC. Epidemiological investigation showed that chronic HBV carriers have a 5- to 15-fold increased risk of HCC compared with the general population [1]. Recent studies have demonstrated that genetic alterations alone cannot account for the complexity of human HBV-related hepatocarcinogenesis, but that epigenetic changes, such as DNA methylation [2], histone modifications [3], and noncoding RNA expression [4, 5], are also involved in this process. The hepatitis B virus X protein (HBx) has been implicated in HBV-related hepatocarcinogenesis and considered to be oncogenic [6, 7]. HBx transgenic mice can develop HCC, further demonstrating that HBx may have independent carcinogenic effects [8]. Therefore, the mechanism by which HBx leads to the transformation from normal liver cells to hepatocarcinoma has attracted widespread interest. There are epigenetic modifications, as much as genetic regulation involved in HBx-induced hepatocarcinogenesis [9].

As a kind of noncoding RNA, long non-coding RNAs (lncRNAs) are characterized by their complexity and the diversity of their sequence characteristics and mechanisms of action [10]. The potential treasure trove of lncRNAs remain unclear and has attracted intense scientific interest, as genome-wide transcriptome studies have revealed that approximately 10- to 20-fold more genomic sequences are transcribed as lncRNAs than as protein-coding RNAs. Recent studies have implicated lncRNAs in a variety of biological functions and pathological processes [11], and altered lncRNA levels can result in aberrant gene expression, which may contribute to cancer biology [12, 13]. Using lncRNA microarrays analysis, we have found that the lncRNA profiles were aberrantly expressed in HBV-related HCC tissues compared with the paired adjacent noncancerous hepatic tissues, and lncRNA-HEIH was an oncogenic lncRNA that promotes tumor progression [5]. These indicate that lncRNAs may serve as key regulatory hubs in HBV-related HCC progression.

In our previous study, we also found that the HBx-transgeneic mice have a different liver lncRNA expression profiles compared with the wild-type mice. We identified an lncRNA, Dreh, which was down-regulated by HBx, can inhibit HCC growth and metastasis acting as a tumor suppressor in the development of HBV-HCC [14]. This discovery contributes to a better understanding of the importance of the deregulated lncRNAs by HBx in HCC. However, the mechanism by which HBx results in the aberrant expression of lncRNAs remains unknown.

In this study, we analyzed the transcriptional factor binding sites in the promoters of the differentially expressed lncRNAs in the livers of HBx-transgenic mice by bioinformatics prediction. Our results indicate that Evi1 (ecotropic viral integration site 1) is a key transcription factor (TF) in the regulatory network of these HBx-induced dysregulated lncRNAs. We further investigated the biological function of EVI1, *in vitro* and *in vivo*, and found that it can promote cell proliferation of HBx-induced hepatocarcinogenesis.

## RESULTS

### Evi1 was predicted as a critical transcription factor in HBx-transgenic mice liver

Since in our previous work, we examined the expression profiles of the livers of the 20-month-old male HBx-transgenic mice and the wild-type mice, including lncRNAs and mRNA profiles (microarray data have been deposited in National Center for Biotechnology Information (NCBI) Gene Expression Omnibus (GEO) under the accession number GSE42185). The results showed that a series of lncRNAs and mRNAs were frequently aberrantly expressed in the livers of HBx-transgenic mice. The differential transcripts expression induced by HBx-overexpression and they may be related to the HBx-induced hepatocarcinogenesis.

**Figure 1:**
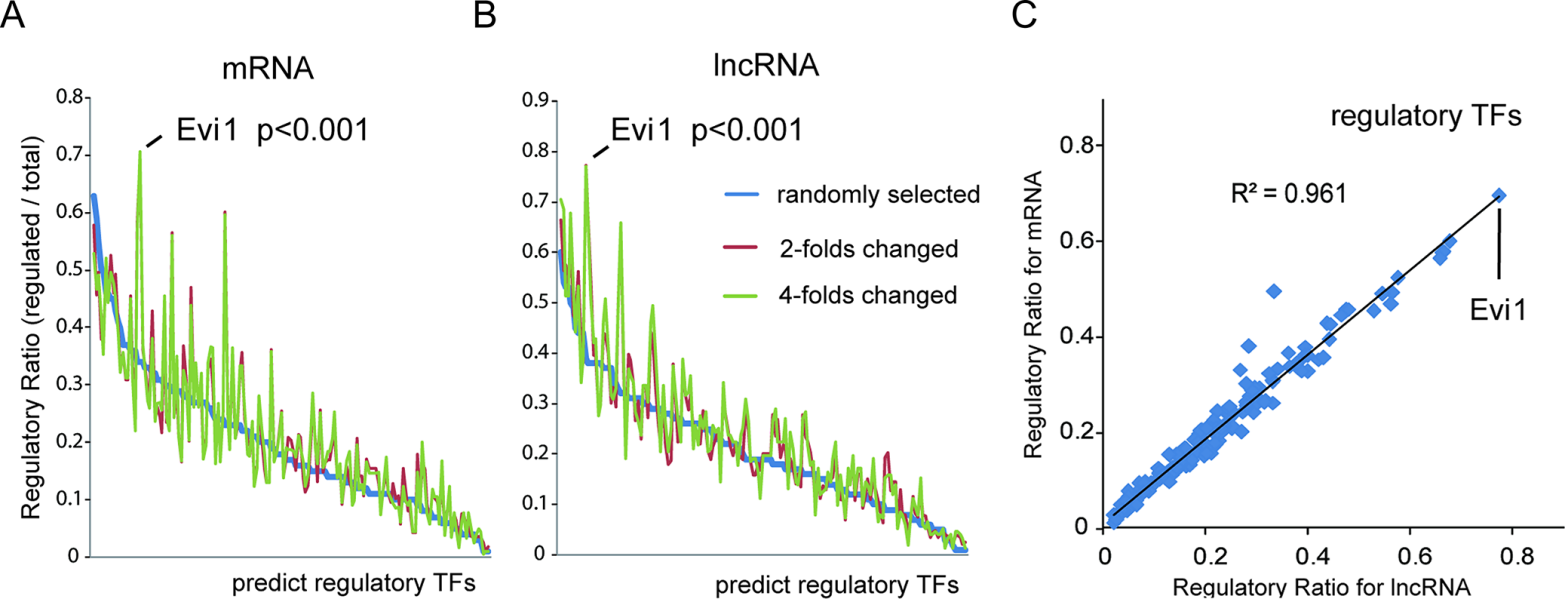
Bioinformatics analysis of critical transcription factors of differentially expressed lncRNAs and mRNA in HBx-transgenic mice livers (**A** and **B**) The potential TFs contributing to differential expression of mRNAs (A) and lncRNAs (B) were predicted with bioinformatics approaches using a well-established promoter-predicted model and a commercial database of TF binding site sequences. The regulatory weight of each TF was calculated by its regulatory ratio, i.e., the count of specific-TF regulated transcripts/the count of all changed transcripts. Transcripts with a 2- or 4-fold change in HBx-transgenic mice livers were chosen for analysis, and 100 randomly selected transcripts were also selected as a background control. Evi1 was shown to be the TF with the highest regulatory ratios for both lncRNAs and mRNAs. (**C**) The regulatory ratios of each TF for those lncRNAs and mRNAs with greater than 2-fold changes were subjected to a Pearson correlation analysis (*R*^2^ = 0.961, *P* < 0.001, Pearson's correlation).

To investigate the mechanism of the upstream regulation of these transcripts, we attempted to identify the common TFs controlling the specific expression of mRNAs and lncRNAs in our HBx-overexpression model. As the transcription factors can bind to specific DNA sequences of promoter regions adjacent to genes and regulate gene expression, we predicted potential TFs that contribute to the differential expression of lncRNAs and mRNAs using bioinformatics approaches, with a well-established promoter-predicted model and a commercial database of TFs binding site sequences. The weight of each TF contributes to the differential expressed genes was also evaluated using the ratio of regulated genes to all changed genes. One hundred randomly selected transcripts were used as a background control. Interestingly, the deregulated lncRNAs and mRNAs shared a very similar profile for predicted regulating TFs, which was significantly different compared with the profile of predicted TFs for the group of randomly selected transcripts (Figure 1A, 1B; Supplementary Tables 1, 2). This finding indicated that a unique cluster of TFs may specifically contribute to the differential expression of both the lncRNAs and mRNAs in the HBx-transgenic mouse liver. And among these potentially HBx-regulated TFs, Evi1 showed the highest ratios in differential transcriptional regulation for both lncRNAs and mRNAs, which may be a key regulatory agent in HBx-induced pathogenesis (Figure 1C).

### Evi1 regulates a cluster of lncRNAs expression as a transcription factor

**Figure 2:**
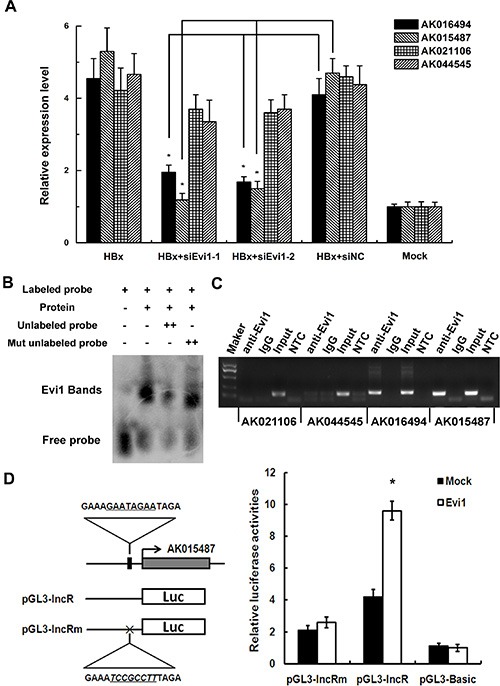
Evi1 regulates a cluster of lncRNAs expression as a transcription factor (**A**) The relative expression of four predicted Evi1 target lncRNAs (AK016494, AK015487, AK021106, and AK044545) in mouse BNL CL.2 cells co-transfected with pEGFP-HBx (HBx) and Evi1-specific siRNA (siEvi1-1 or siEvi1-2) by qRT-PCR. (**B**) EMSA shows that Evi1 protein can bind to the promoter of lncRNA-AK015487. The specificity of binding was examined by competition with the unlabeled probes. (**C**) The binding activity of Evi1 at the promoters of AK016494, AK015487, AK021106, and AK044545 in pEGFP-HBx transfected BNL CL.2 cells was evaluated with ChIP assays. Rabbit total IgG was used as a negative control. For PCR assays, non-template control (NTC) was also used to evaluate the primers. (**D**) Dual luciferase assay of BNL CL.2 cells cotransfected with the reporter vectors containing wild-type (pGL3-lncR) or mutated (pGL3-lncRm) predicted Evi1-binding sites of lncRNA AK015487 promoter. A mock vector (pGL3-Basic) was used as a control. *P < 0.05.

As shown in our previously predicted data, among the transcription factors implicated in HBx-transgenic mice, Evi1 appears to have a great influence on the regulation of mRNAs and lncRNAs. As one of the most studied regulatory factor, Evi1 contains DNA-binding domains, which attach to specific sequences of DNA adjacent to the genes that they regulate, then influence the transcriptional activity of many protein encoding genes, such as Smad3, Cebpe and Serpinb2 [15, 16]. In order to confirm if Evi1 was also a key TF involved in regulating HBx-related lncRNAs, four predicted Evi1 target lncRNAs (AK016494, AK015487, AK021106, and AK044545) which obviously up-regulated in HBx-transgenic mice compared with wild-type mice were further examined for their expression levels and the Evi1 target sites with qRT-PCR and ChIP assays in HBx-overexpressing mouse hepatocytes. The expression levels of lncRNAs were evaluated with qRT-PCR in Evi1-inhibition hepatocytes subsequent to HBx-overexpressing. The results indicated that the four lncRNAs were all up-regulated in HBx-overexpressing mouse BNL CL.2 cells and the elevation of AK016494 and AK015487 were greatly abolished by co-transfection of Evi1-specific siRNAs (Figure 2A). The gene-silencing efficiency of Evi1 siRNA compared with the negative control siRNA was shown in Supplementary Figure 1. In ChIP assays, we observed enrichment of lncRNAs AK016494 and AK015487's promoters using the Evi1 antibody versus a nonspecific antibody (IgG control) (Figure 2C). To further validate the Evi1 binding sites in the promoter region of lncRNA, we performed promoter analysis of lncRNA AK015487 using luciferase reporter assays. The promoter sequences of AK015487 including the predicted Evi1-binding sites, with or without mutations, were cloned into the pGL3 plasmid and cotransfected with the mouse Evi1 expressing vector or negative control vector in BNL CL.2 cells. As shown in Figure 2D, Evi1 significantly increased the luciferase activity of the wild-type construct of AK015487 promoter with respect to the negative control, whereas such an enhanced effect was not observed in cells with the mutated construct of AK015487 promoter.

We further tested the ability of the Evi protein to bind to the promoter of lncRNA-AK015487 using an electrophoretic mobility shift assay (EMSA). A DNA fragment containing the predicted Evi1-binding sites of AK015487's promoter region was labeled using biotin and used as a probe. The results showed that no binding signal was detected in reactions without recombinant protein (Figure 2B, lane 1); but when incubated the labeled probe with the recombinant protein, a retarded DNA-protein complex was detected (Figure 2B, lane 2). In addition, competition with excess unlabelled DNA fragment reduced the amounts of complexes (Figure 2B, lanes 3), whereas the mutant competitor DNA was unable to abolish the binding of the protein to the probe (Figure 2B, lanes 4). These results all indicate that as a regulatory factor, Evi1 can interact with the promoters of lncRNAs, such as AK015487, and regulate their transcriptional activity.

### Evi1 is highly expressed in HBx-transgenic mice and HCC cell lines expressing HBx

**Figure 3:**
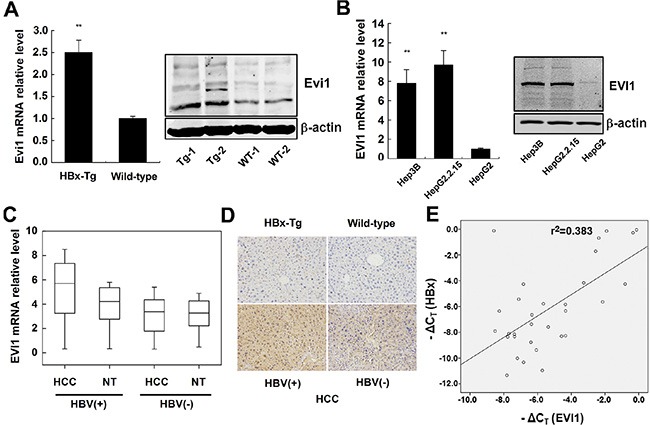
EVI1 was expressed at high levels in HBx-transgenic mice, HCC cells expressing HBx and human HBV-related HCC tissues (**A**) The relative mRNA and protein expression of Evi1 in HBx-transgenic mice comparison to wild-type mice livers. (**B**) Relative expression of EVI1 in Hep3B, HepG2.2.15, and HepG2 cells. Data are shown as means and standard deviations from triplicate experiments. **P < 0.01. (**C**) EVI1 mRNA expression in HCC tissues versus paired adjacent noncancerous hepatic tissues (from 31 pairs of HBV-related HCC patients and 30 pairs of HBV-negative HCC patients). Statistical differences between HBV-HCC tissues and the paired adjacent noncancerous hepatic tissues were analyzed with the paired-samples *t* test (*P* < 0.0001). (**D**) Immunohistochemistry staining of Evi1/EVI1 protein in the livers of HBx-transgenic mice and the wild-type mice, patient samples of HBV-related and HBV-negative HCC tissues. (**E**) The EVI1 and HBx expression levels were positively correlated in 31 pairs of HBV-related HCC samples. EVI1 and HBx expression levels were determined by real-time PCR and normalized to β-actin, and respective ΔC_T_ values were subjected to a Pearson correlation analysis (*r* = −0.619, *P* < 0.01, Pearson's correlation).

To investigate the relationship between HBx and the Evi1 expression, we first assessed the mRNA and protein expression levels of mouse Evi1 in both HBx-transgenic and wild-type mice livers by qRT-PCR and western blot analysis. The results showed that Evi1 was significantly up-regulated in HBx-transgenic mice livers compared with the wild-type mice (Figure 3A). Immunohistochemistry staining analysis of mouse liver samples showed the same results (Figure 3D, upper panel).

Next, we determined whether EVI1 was expressed differently in human HCC cells. The expression of EVI1 mRNA and protein were markedly higher in the Hep3B (a cell line containing the integrated hepatitis B viral genome) and HepG2.2.15 (a derivative of the human hepatoma cell line HepG2 that has been stably transformed with a head-to-tail dimer of HBV DNA) cell lines versus HepG2 cells which doesn't expressing HBx (Figure 3B).

### The mRNA expression level of EVI1 is up-regulated in HBV-related HCC tissues

To further investigate whether EVI1 was expressed differentially in human primary liver cancer, we measured EVI1 mRNA and protein expression levels in 31 pairs of human HBV-related HCC tissues and 30 pairs of HBV-negative HCC tissues and their pair-matched normal liver tissues by real-time PCR and IHC staining. The results showed that the expression levels of EVI1 were significantly up-regulated in HBV-HCC tissues in comparison with the adjacent noncancerous hepatic tissues from the same patient (*P* < 0.0001, paired-samples *t* test), however, no significant difference was observed in the expression levels between the HBV-negative HCC tissues and the adjacent noncancerous hepatic tissues (Figure 3C). Furthermore, the qRT-PCR and immunohistochemistry staining also showed that the expression of EVI1 mRNA and protein were significantly higher in HBV-positive HCC tissues compared with the HBV-negative HCC tissues (Figure 3C; Figure 3D, lower panel).

### HBx and EVI1 mRNA levels are positively correlated in HBV-related HCC tissues

Next, we asked whether the increased EVI1 expression was correlated with the levels of HBx expression in human HBV-related HCC tissues. We further analyzed the expression levels of HBx in the aforementioned thirty-one HCC tissues. A statistically significant positive correlation was observed between EVI1 and HBx mRNA (*r* = −0.619, *P* < 0.01, Pearson's correlation; Figure 3E). These data suggested the potential positive regulation of EVI1 expression induced by HBx in human HCCs, and suggested that EVI1 might be involved in HCC pathogenesis as a regulatory TF subsequent to HBx overexpression in chronic hepatitis B patients.

### Enforced HBx expression up-regulates EVI1 in both mouse and human liver cells

**Figure 4:**
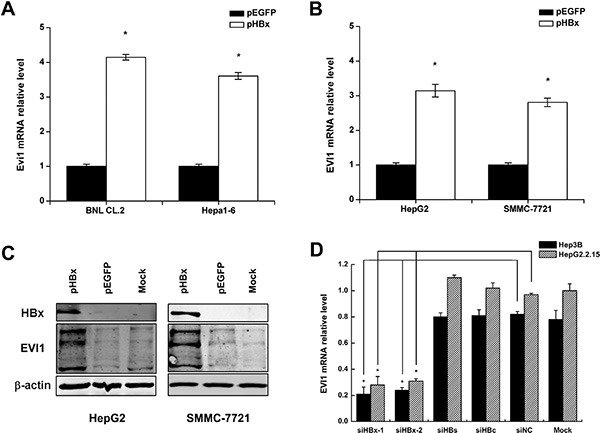
Enforced HBx expression up-regulates EVI1 in both mouse and human liver cells (**A**) Mouse Evi1 mRNA expression after the transfection of pEGFP-HBx or the control pEGFP plasmid in mouse BNL CL.2 and Hepa1-6 cells. (**B**) Human EVI1 mRNA expression after the transfection of pEGFP-HBx or the control pEGFP plasmid in human HepG2 and SMMC-7721 cells. (**C**) Western blot analysis of human EVI1 protein expression in HepG2 and SMMC-7721 cells transfected with pEGFP-HBx or respective controls, and the no treatment control cells (mock). (**D**) Human EVI1 mRNA levels after the transfection of HBx-specific siRNA (siHBx-1 or siHBx-2) or HBs-specific siRNA or HBc-specific siRNA or control siRNA (siNC) in Hep3B and HepG2.2.15 cells. Data are shown as means and standard deviations from at least three independent experiments. *P < 0.05.

To investigate whether HBx alters EVI1 expression, we measured the levels of mouse Evi1 mRNA after the transient transfection of pEGFP-HBx into mouse liver cell lines, including BNL CL.2 and Hepa1-6 cells. We found that EVI1 was up-regulated in pEGFP-HBx-transfected cells in comparison with the pEGFP control groups (Figure 4A).

To confirm this phenomenon in human, we measured the human EVI1 mRNA levels and protein expression in HCC cells (HepG2 and SMMC-7721) after the transient transfection of pEGFP-HBx and the control vectors, and the results showed the same in human (Figure 4B), western blot analysis also showed EVI1 protein was increased by enforced HBx expression in HepG2 and SMMC-7721 cells (Figure 4C). EVI1 antibody detects endogenous levels of total EVI1 and MDS1/EVI1 proteins. The three bands from top to bottom showed in EVI1 western blot represent MDS/EVI1, EVI1 and EVI1Δ respectively. MDS/EVI1 denotes the fusion between MDS1 and EVI1 [17], and EVI1 delta denotes the truncated form of EVI1 [18]. On the contrary, the inhibition of HBx by siRNA decreased the EVI1 mRNA expression in HepG2.2.15 and Hep3B cells which have HBx expressing, and the inhibition of other HBV components like HBs or HBc didn't have the same effect. (Figure 4D).

### Inhibition of EVI1 suppresses cell proliferation of HCC cells expressing HBx *in vitro*

**Figure 5:**
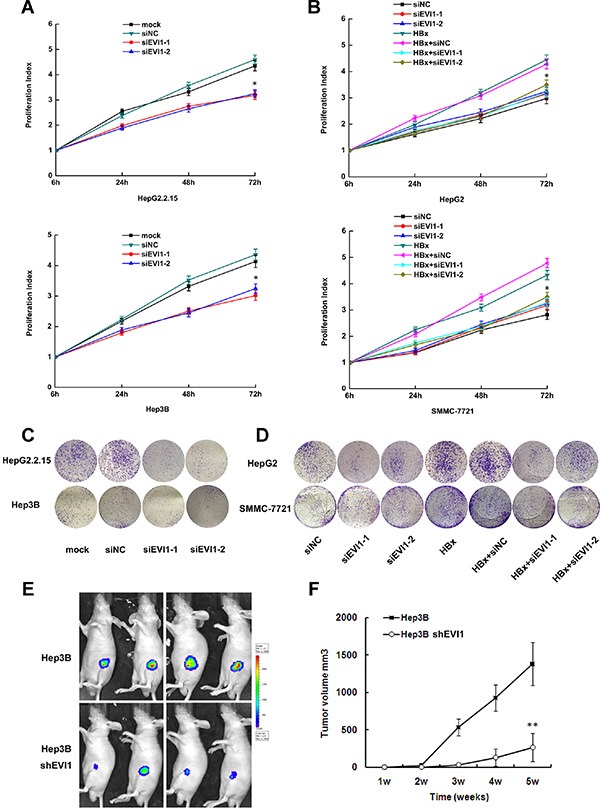
Inhibition of EVI1 suppresses cell proliferation of HCC *in vitro* and *in vivo* (**A** and **C**) Proliferation activity of HepG2.2.15 and Hep3B cells after transfected with siRNA against EVI1 or respective controls using the CCK8 assay and the colony formation assay. (**B** and **D**) Proliferation activity HepG2 and SMMC-7721 cells after co-transfected with pEGFP-HBx and Evi1-specific siRNA (siEvi1-1 or siEvi1-2) or control siRNA (siNC) using the CCK8 assay and the colony formation assay. For CCK8 assay, cells were seeded in 96-well plates, and OD 450 nm were assessed 6, 24, 48, and 72 hours after cells were adherent. Data are shown as the mean ± SD based on at least three independent experiments. *P < 0.05. (**E**) Photographs of tumors that developed in nude mice (Hep3B with shRNA against EVI1 or the control shRNA) by imaging with the IVIS Imaging System (Caliper Life Sciences, Hopkinton, MA), four weeks after cell injection. (**F**) Tumor volume in the two groups of nude mice after ectopic-subcutaneous implantation of Hep3B cells transfected with shRNA-EVI1 or the control shRNA. Data are the mean ± SD. *P < 0.05. **P < 0.01.

All the results above imply that EVI1 may have a role in HBV-related hepatocarcinogenesis. To prove this, the effects of repressed expression of EVI1 on cell proliferation, apoptosis and cell migration were investigated in several human HCC cell lines expressing HBx. We repressed the EVI1 expression by RNA interference. The results showed that EVI1 had little effects on the migration or apoptosis of hepatocytes (data not shown). Cell-counting kit-8 assays and colony formation assays indicated that cell proliferation were reduced in both HBx expressing cell lines HepG2.2.15 and Hep3B when EVI1 expression was knocked down by EVI1 specific siRNAs compared with the negative control siRNAs (Figure 5A, 5C).

We further transfected pEGFP-HBx plasmid to mock the enforced expressing of HBx in HepG2 and SMMC-7721 cells, which didn't express HBx naturally. Consistent with the above results, co-transfection with EVI1 specific siRNAs also showed reduced proliferation compared with cells transfected with pEGFP-HBx only in these two HCC cells (Figure 5B, 5D). These results suggested that EVI1 may play a key role in HBx induced hepatocellular proliferation.

### Inhibition of EVI1 suppresses the growth of tumor *in vivo*

To determine the effects of EVI1 on tumorigenesis *in vivo*, EVI1-down-regulated or control cells (firefly luciferase-labeled Hep3B cells stably expressing either shRNA-EVI1 or control shRNA) were subcutaneously injected into the armpit of nude mice for xenoplantation. We observed that mice injected with cells transfected with shRNA-EVI1 showed significantly decreased tumor growth compared with those injected with cells transfected with control shRNA (Figure 5E, 5F). These results further indicated that EVI1 was involved in the biological function of cell proliferation in HBV-related HCC.

### Overexpression of lncRNA-AK015487 partially recovers the cell proliferation activity suppressed by Evi1 inhibition in mouse hepatoma cells expressing HBx

To investigate whether the Evi1 target lncRNAs are also involved in HBx-induced hepatocarcinogenesis, we first performed a rapid amplification of cDNA ends (RACE) analysis to identify the 5′ and 3′ ends of the transcript to get the full-length cDNA of lncRNA-AK015487, then the full length cDNA was sub-cloned into a pcDNA3.1 plasmid to construct an expression vector pcDNA3.1-AK015487. The transcription start and termination sites and sequences of full-length cDNA of AK015487 are presented in Supplementary Figure 2.

**Figure 6:**
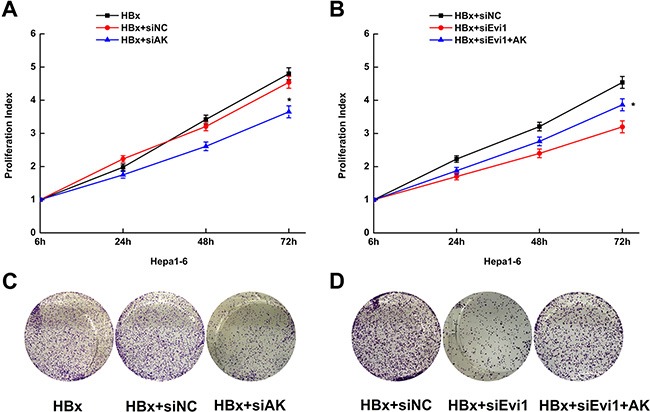
Overexpression of lncRNA-AK015487 partially recovers the cell proliferation activity suppressed by Evi1 inhibition in mouse hepatoma cells expressing HBx (**A** and **C**) Proliferation activity of Hepa1-6 cells after co-transfected with pEGFP-HBx and AK015487-specific siRNA (HBx+siAK) or control siRNA (HBx+siNC) using the CCK8 assay and the colony formation assay. (**B** and **D**) Proliferation activity of Hepa1-6 cells co-transfected with AK015487 expression vector, Evi1 specific siRNAs and pEGFP-HBx (HBx+siEvi1+AK); control pcDNA3.1 vector, Evi1 specific siRNAs and pEGFP-HBx (HBx+siEvi1); control siRNA and pEGFP-HBx (HBx+siNC) using the CCK8 assay and the colony formation assay. Data are shown as the mean ± SD based on at least three independent experiments. *P < 0.05.

The effects of AK015487 on cell proliferation were evaluated in mouse hepatoma cell line (Hepa1-6 cell) expressing HBx by cell-counting kit-8 assays and colony formation assays. Cells were co-transfected with HBx expression plasmid and lncRNA-AK015487 specific siRNAs or negative control siRNAs. The results showed that the inhibition of AK015487 by siRNA suppressed HBx-induced cell proliferation just like the siRNA-Evi1 (Figure 6A, 6C).

Moreover, we upregulated the AK015487 expression by transfection of pcDNA3.1-AK015487 plasmid together with the Evi1 specific siRNAs in HBx-enforced expressed Hepa1-6 cells. And found the reduction of cell proliferation induced by Evi1 inhibition was partially recovered by co-transfection of AK015487 expression vector (Figure 6B, 6D). The rescue effect by overexpression of AK015487 indicated that lncRNA-AK015487 is involved in Evi1-induced cell proliferation activation in HBx-related hepatocarcinogenesis.

## DISCUSSION

LncRNAs are becoming one of the hot topics in genome research, with the alluring possibility of advancing our comprehensive understanding of biological processes in human health and diseases. Recent studies have revealed the various functions and molecular mechanisms of these enigmatic molecules [10, 11, 12], therefore, more and more researchers participate in these area. With the development of high-throughput detection technology, such as lncRNAs microarray and RNA sequencing, the aberrantly expressed lncRNAs profiles of a large number of various diseases were performed [22, 23]. However, although the number of lncRNAs is a lot more than mRNAs, the role of most of lncRNAs is unknown, and it is evident that there are many functional large non-coding RNAs. But the methods to study the function and mechanism of lncRNAs are rare. Two common mechanisms of lncRNAs function widely reported by now are: interfere with the transcription efficiency of downstream gene [24] or antisense gene [25, 26], interact with the RNA-binding proteins [27]. In this paper, we provide a new approach to predict the functions of the aberrantly expressed lncRNAs.

That is, through transcription factor analysis, further investigation of the roles of the TFs and their regulated mRNAs will suggest the functions of these lncRNAs. The roles of the TFs-regulated mRNAs could be examined by various methods, such as gene ontology (GO) and pathway analysis. Our results confirmed that the differentially expressed lncRNAs and mRNAs shared a very similar predicted regulating TFs profile in HBx-transgenic mice livers. Among them, Evi1 showed the highest ratio and was predicted as a critical transcription factor. Thus, our study developed a potential new approach to investigate the function of lncRNAs.

As we known, many diseases have genetic heterogeneity, the great individual differences of human diseases coupled with the ethical issues that may arise, result in that the pathogenic mechanism and molecular function studies of many diseases cannot be carried out in humans, We can only performed these studies in animal models. A large number of experiments and studies have performed and found the dysregulated lncRNAs profiles of various diseases in mouse models [28–30]. However, an important different feature between lncRNAs and protein-coding genes are in the evolutionary conservation [10, 31]. Many lncRNAs are poorly conserved and lack of interspecies sequence conservation [32]. Since the huge species differences of lncRNAs expression, how to use the results found in the animal models for the mechanisms research and clinic of human diseases is a problem. While in transcription factors, such as Evi1, such phenomenon does not exist, they have very high interspecies homology. As described previously in this paper, the pathological process about Evi1 found in HBx-transgenic mice initially has been verified in human HBx-induce hepatocarcinogenesis. Therefore, our work has enormous contribution and significance to translate the research results of lncRNAs from animal models into human disease studies and clinical practice.

Furthermore, we identified a critical transcription factor, EVI1, which plays a key role in HBx-induced hepatocarcinogenesis acting as an oncogene, and the inhibition expression of EVI1 can suppress cell proliferation of HCC *in vitro* and *in vivo*. EVI1 has long been known as one of the dominant oncogenes associated with murine and human myeloid leukemia [33, 34]. In recent years, EVI1 was also found to contribute to colorectal cancer [35] and breast cancer [36]. However, whether and how EVI1 plays a role in HCC development remains largely unknown. In this present study, with bioinformatics analyses and loss-of-function approaches, EVI1 was identified as a key regulatory TF subsequent to HBx overexpression. The TF was up-regulated in HBx-transgenic mice and human HCC cells and contributed to hepatocyte proliferative activity. In this regard, we suggest that EVI1 may be also an oncogene involved in HBV-related HCCs. It has been known that EVI1 regulates multiple cellular processes important for cancer [37]. The EVI1 can repress the transforming growth factor-β (TGF-β) signaling and antagonize the growth-inhibitory effects of TGF-β by interactions with Smad3, an intracellular mediator of TGF-β signaling [15, 35]. It can also increase AP-1 activity and stimulate c-fos promoter transactivation, which may also contribute to the aberrant expression of oncogenes [38]. These EVI1 affected signaling pathways may also have potential relationship with HBx-induced phenotypic changes. The HBx protein has been implicated in HBV-related HCC pathogenesis acting as a transcriptional activator, which can enhance the activation of transcription factors, such as AP-1 and NF-kappa B [39, 40]. Our findings about EVI1 are an important supplement to the HBV-HCC tumorigenesis and transcription factor regulation network. And in our experiments, transfection of EVI1-sepical small interfering RNA decreased the proliferation of HCC cells overexpressing HBx, and suppressed the tumor growth *in vivo*. Which mean that, the inhibition of EVI1 expression could reverse the malignant phenotypes of HCC cells overexpressing HBx. This may provide a potential therapeutic target for the prevention and treatment of HCC.

## MATERIALS AND METHODS

### Animal and patient samples

The HBx transgenic mice were constructed by the Model Animal Research Center of Nanjing University (Nanjing, China). The 4-weeks old male BALB/C nude mice used in this study were purchased from the Shanghai Experimental Animal Center of the Chinese Academy of Sciences (Shanghai, China). All mice were bred and maintained in a pathogen-free facility and were used in accordance with the institutional guidelines for animal care. The animal studies were approved by the Institutional Animal Care and Use Committee of the Second Military Medical University, Shanghai, China.

The 31 HBV-related HCC tissues and 30 HBV-negative HCC tissues and the corresponding nearby noncancerous liver tissues used in this study were obtained with informed consent from patients who underwent radical resections in the Eastern Hepatobiliary Surgery Hospital (Second Military Medical University, Shanghai, China). Studies using human tissues were reviewed and approved by the Committees for Ethical Review of Research involving Human Subjects of Second Military Medical University (Shanghai, China).

### Transcription factor binding site prediction

Here, we present a bioinformatics analysis strategy to predict TF binding sites in the promoters of both the lncRNAs and mRNAs whose expression was changed by 2- or 4-fold in the livers of HBx-transgenic mice compared with wild-type mice. We also randomly selected 100 lncRNAs and 100 mRNAs (shown in Supplementary Table 3) for a background analysis of TF binding sites. The detailed methods are supplied in the Supplementary Materials and Methods. In brief, we first identified the transcription start sites (TSS) of all of the differentially expressed genes according to the ECRbase website http://ecrbase.dcode.org/promoters.php and then predicted their promoters as the regions −1,500 bp to 200 bp from the TSSs of individual genes [19]. The sequence of the genes' promoter regions were examined for TF binding sites using TRANSFAC Professional version 8.1 and the HMMER package (version 2.2) [20].

### Construction of vectors

The plasmid included green fluorescent protein pEGFP-HBx vector was constructed in our laboratory previously [21]. For lncRNA AK015487 promoter constructor (pGL3-lncR), a 1.5-kilobase pair promoter fragment of the lncRNA AK015487 and the promoter region containing mutation in the Evi1 binding site (from GAAAGAATAGAATAGA to GAAATCCGCCTTTAGA) were inserted up stream of the luciferase gene in pGL3-Basic vector. The mutant in the Evi1-binding site was generated by overlap extension method. To construct Evi1 expressing vector, the complementary DNA encoding mouse Evi1 was PCR-amplified and sub-cloned into the pcDNA3.1 vector (Invitrogen, Carlsbad, CA). The AK015487 expression vector pcDNA3.1-AK015487 contained the full length cDNA sequences, which was amplified by PCR and sub-cloned into a pcDNA3.1 vector. All vectors were constructed according to standard methods and verified by sequencing.

### Cell culture and transfection

The liver cell lines HepG2, HepG2.2.15, Hep3B, SMMC-7721 and BNL CL.2, Hepa1-6 were obtained from the American Type Culture Collection. The cells were grown in Dulbecco's Modified Eagle Medium (Gibco BRL) with 10% fetal bovine serum (Gibco BRL) and were maintained in an atmosphere of 5% CO_2_ in a humidified 37°C incubator. Transfections were performed using the Lipofectamine 2000 kit (Invitrogen, Carlsbad, CA) according to the manufacturer's instructions. The specific siRNA and negative control RNA (GenePharma) were introduced into cells at a final concentration of 20 nM. The transfected cells were harvested at 24, 48, or 72 hours after transfection. The siRNAs were synthesized by GenePharma (Shanghai, China) as described. The knockdown efficacy of siRNAs was shown in Supplementary Figure 1. The sequences are depicted in Supplementary Table 4.

### Reverse transcription and quantitative real-time PCR

Total RNA was extracted using the Trizol reagent (Takara, Dalian, China). First-strand cDNA was generated using the Reverse Transcription System Kit (Stratagene, La Jolla, CA). Real-time PCR was performed using a standard SYBR-Green PCR kit protocol in a StepOne Plus system (Applied Biosystems, Foster City, CA). β-actin was employed as an endogenous control to normalize for the amount of total mRNA in each sample. The real-time PCR reactions were performed in triplicate. The relative RNA expression was calculated using the comparative Ct method. The primer sequences are presented in Supplementary Table 4.

### Chromatin immunoprecipitation assay

Chromatin immunoprecipitation (ChIP) were performed using the EZ ChIP™ Chromatin Immunoprecipitation Kit (Millipore Bedford, MA, USA) according to its manual. Briefly, cross-linked chromatin was sonicated into 200- to 1000-bp fragments. The chromatin was immunoprecipitated using an anti-Evi1 antibody (ab28457, Abcam). Normal rabbit IgG was used as a negative control. Quantitative PCR was conducted using a standard SYBR-Green PCR kit protocol on a StepOne Plus System (Applied Biosystems, Foster City, CA). The primer sequences are listed in Supplementary Table 4.

### Luciferase reporter assay

Cells (1 × 10^5^) were transfected with 500 ng of Evi1 target construct (pGL3-lncR) or control plasmids (pGL3-lncRm and pGL3-Basic). Each plasmid was cotransfected with 50 ng of pRL-TK plasmid expressing renilla luciferase to monitor the transfection efficiency (Promega). A luciferase activity assay was performed 48 hours after transfection with the dual luciferase reporter assay system (Promega). The relative luciferase activity was normalized with renilla luciferase activity.

### Electrophoretic mobility shift assay

Electrophoretic mobility shift assay (EMSA) was performed using the LightShift™ Chemiluminescent EMSA Kit (Thermo Fisher Scientific) according to its manual. Binding reactions were performed by adding the recombinant Evi1 protein (Cusabio Biotech, China) to a mixture binding buffer containing biotin-labeled, double-stranded probes, which include the predicted Evi1-binding sites. The specificity of binding was examined by competition with the unlabeled probes. Competition reaction mixtures contained a 100-fold molar excess of unlabeled double-stranded probes. The mixtures were then resolved by nondenaturing PAGE and transferred to a nylon membrane, finally visualized by horseradish peroxidase-conjugated streptavidin. The probe sequences are listed in Supplementary Table 4.

### Western blot analysis

Cell lysates were prepared with RIPA buffer (Pierce). Identical protein quantities were separated by sodium dodecyl sulfate-polyacrylamide gel electrophoresis and transferred onto polyvinylidene fluoride membranes. After incubation with antibodies specific for mouse Evi1 and human EVI1 (1:1000, Cell Signaling Technology), HBx (1:1000, Abcam) or β-actin (1:5000, Sigma-Aldrich), the blots were incubated with IRdye 800-conjugated goat anti-rabbit IgG or IRdye 700-conjugated goat anti-mouse IgG (1:10000, Rockland Immunochemicals, Gilbertsville, PA). Immunofluorescence was detected using an Odyssey infrared scanner (Li-Cor, Lincoln, NE).

### Immunohistochemistry staining

For immunohistochemical staining assay, paraffin-embedded mouse and human liver samples were sectioned at a thickness of 4–5 μm and placed on clean slides. After blocking with goat sera, immunohistochemistry was performed with polyclonal anti-EVI1 antibodies (ab28457, Abcam). The slides were photographed with a Zeiss axiophot photomicroscope (CarlZeiss, Oberkochen, Germany). Five pictures on each slide were randomly taken. The integrated optical density (IOD) of pictures was measured using Image Pro Plus 6.0 software.

### Measurement of cell proliferation

Cells (2 × 10^3^ cells/well) were dispensed in 100 μl aliquots into 96-well plates. At the indicated time points, the Cell Counting Kit-8(Dojindo, kumamoto, Japan) was added to the cells for 2 hours and then the optical density was read using a microplate reader (BIO-RAD, Hercules, CA). All of the experiments were performed in triplicate.

For colony formation assay, cells were seeded at a density of 100 cells per well in a 12-well culture plate and cultured for 2 weeks, the colonies were stained with 1% crystal violet and counted.

### *In vivo* tumorigenesis assay

Lentivirus-based shRNA constructs (GenePharma, Shanghai, China) were used to stably knock-down EVI1 gene expression according to manufacturer's instructions. Hep3B cells (firefly luciferase-labeled) were stably transducted with EVI1 shRNA lentivirus. Cells transfected with EVI1 shRNA and the control cell lines (1.0 × 10^7^) were implanted subcutaneously into the flanks of nude mice. Primary tumor growth was analyzed with the *in vivo* imaging system using the IVIS Lumina II (Caliper Life Sciences, Hopkinton, MA) 10 min after intraperitoneal injection of 4.0 mg of luciferin (Gold Biotech) in 50 μl of saline, and tumor volumes were calculated according to the equation V = 0.4 × LW^2^.

### 5′ and 3′ rapid amplification of cDNA ends

We used the 5′-RACE and 3′-RACE analyses to determine the transcriptional initiation and termination sites of lncRNA-AK015487 using a SMARTer RACE cDNA Amplification Kit (Clontech, Palo Alto, CA) according to the manufacturer's instructions. The gene-specific primers used for the PCR of the RACE analysis are presented in Supplementary Table 4.

### Statistical analysis

The expressions of EVI1 in HCC patients were compared by the paired-samples *t* test. The relationship of EVI1 and HBx mRNA expression was analyzed by Pearson's correlation. Others comparisons were determined by Student's *t*-test. All *P* values were two-sided and obtained using the SPSS 18.0 software package (SPSS, Chicago, IL, USA). Differences were defined as statistically significant for *p*-values < 0.05.

## SUPPLEMENTARY MATERIALS FIGURES AND TABLES



## Abbreviations

HBV: hepatitis B virus
HBx: hepatitis B virus X protein
HCC: hepatocellular carcinoma
lncRNA: long non-coding RNA
EVI1: ecotropic viral integration site 1
TF: transcription factor
qRT-PCR: quantitative reverse transcription-polymerase chain reaction
TSS: transcription start sites
siRNA: small-interfering RNA
ChIP: chromatin immunoprecipitation
EMSA: electrophoretic mobility shift assay
IHC: immunohistochemistry
RACE: rapid amplification of cDNA ends
