# Supplementary material for: EVI1 promotes cell proliferation in HBx-induced hepatocarcinogenesis as a critical transcription factor regulating lncRNAs
Source: Oncotarget. 2016 Mar 8;7(16):21887–99. doi: 10.18632/oncotarget.7993 (PMC5008331; doi:10.18632/oncotarget.7993)
Supplement: Supplementary file 1 [file oncotarget-07-21887-s001.pdf]

EVI1 promotes cell proliferation in HBx-induced hepatocarcinogenesis as a critical transcription factor regulating lncRNAs

Supplementary Materials

MATERIALS AND METHODS

Transcriptional factors prediction

The transcription start sites (TSS) of all the differential genes were identified according to the website ECRbase http://ecrbase.dcode.org/promoters.php [1] and then predicted their promoters by the regions of −1,500 bp~200 bp from the TSSs of individual genes. The sequence information of genes' promoter regions was subjected for TFs binding sites prediction with TRANSFAC Professional version 8.1 and the HMMER package (version 2.2 August 2001) [2].

The flat files of TRANSFAC Professional version 8.1 were parsed to extract two types of alignments: nucleotide sequences used to generate the TRANSFAC NWMs and the nucleotide sequences referenced in the description of the TRANSFAC factors (see below). The vast majority of matrix entries in TRANSFAC lists the accession numbers of the factor(s) associated with that matrix (multiple factors are usually orthologs from different organisms) and the accession numbers of the nucleotide sequences used to generate the matrix referred to below as "sites". Moreover, for each factor TRANSFAC lists which organism the factor belongs to and the accession numbers of the sites described for the factor in target genes. One factor can be linked with more than one matrix, and more than one matrix can describe the same factor. Not all matrices have associated site identifiers, and, more importantly, not all factors that have associated sites were used to build NWMs. Therefore, to extract the maximum amount of information, the TRANSFAC files were parsed following not only the links from "matrices" to "sites" but also the links from "matrices" to "factors" and from there to "sites". We called the alignments retrieved following the links from "matrices" to "sites" matrix-derived alignments. These were optimal multiple sequence alignments that were used as such to build HMMs called matrix derived models and having accession numbers starting with "M". Nucleotide sequences retrieved following the links from "matrices" to "factors" and from there to "sites" were first processed in order to extract the underlying motif using the MEME program downloaded from. For each set of sequences, the MEME search was conducted separately on the forward and on the forward and reverse strands and the best motif was selected taking into account its length and *E*-value; this selection was also verified by manual curation. The resulting MEME alignments, called factor-derived alignments, were used to build HMMs called factor-derived models that have accession numbers starting with "T".

Profile Hidden Markov models were generated using the HMMER package (version 2.2 August 2001). The null model used to generate the models employed equal probabilities for all four nucleotides and took into account the fact that TFBSs can occur frequently throughout the sequence scanned. Therefore we used in the null model a p1 value for the G→G transition controlling the expected length of the target sequences equal to 0.98 instead of the default value of 0.999, thus assuming that two sites for the same TF may occur 50 bp and not 1000 bp apart as in the default model. This significantly decreased the likelihood of retrieving true positive hits with negative scores. The HMMER function hmmpfam searches a sequence or a database of sequences against a library of HMM models, and characterizes each hit it returns by two parameters: the score and the *E*-value. The score is the logarithm in base 2 of the ratio P (seq|HMM) / P (seq|null), where P (seq|HMM) is the probability of the target sequence BMC Bioinformatics 2005, 6:79 http://www.biomedcentral.com/ 1471-2105/6/79 Page 16 of 20 (page number not for citation purposes) according to the HMM model and P (seq|null) is the probability of the sequence according to a null model distribution. The greater the score the better the match between the hit and the model is. The *E*-value, computed with respect to the number of the sequences in the database queried, is a measure of the expected number of false positives that will have scores equal to or larger than the score of the hit. The smaller the *E*-value, the more significant the hit is.

REFERENCES

1. Loots G, Ovcharenko I. ECRbase: database of evolutionary conserved regions, promoters, and transcription factor binding sites in vertebrate genomes. Bioinformatics. 2007; 23:122–124.

2. Madera M, Gough J. A comparison of profile hidden Markov model procedures for remote homology detection. Nucleic Acids Res. 2002; 30:4321–4328.

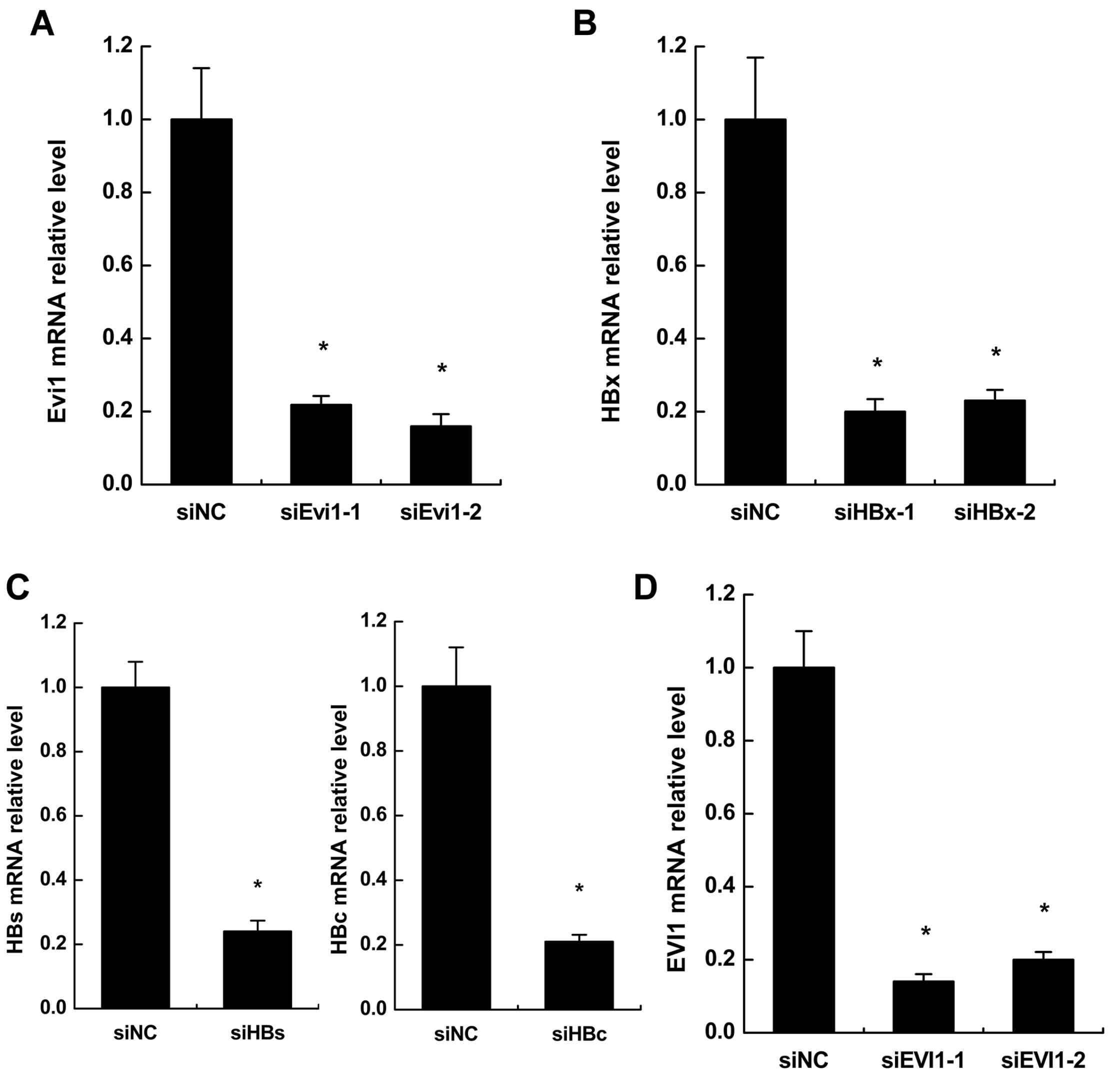

Supplementary Figure S1: The gene-specific silencing efficacy of siRNAs used in this study. (**A**) The relative mRNA expression of mouse Evi1 in mouse BNL CL.2 cells after transfected with siRNA against Evi1 or respective controls using qRT-PCR. (**B**) The relative mRNA expression of HBx in Hep3B cells after transfected with siRNA against HBx or respective controls. (**C**) The relative mRNA expression of HBs and HBc in Hep3B cells after transfected with siRNA against HBs or HBc or respective controls. (**D**) The relative mRNA expression of EVI1 in Hep3B cells after transfected with siRNA against EVI1 or respective controls. Data are shown as means and standard deviations from at least three independent experiments. $*P < 0.05$.

```
   1  GGGGCGCTCC TTAGACAACA TGGCGGGAGG GAGAAAGCGT CTCAGGGGTG AGGCACTCCA
  61  GCGCTTTGCT GGGCGGACAC ATGAAGCTTC CTTTCTCCCG CAGCTCTGTA GAAAAAGGCT
 121  CAACCGATCC CAACCATTCG AAGGAATCCG TTGGATGAGT GGGAGGCCTT GCGTTTATTA
 181  GACTCCCTCC AGTGTAGTCG GAACAAGTGT TCGTTTTACT TTCTTCAGTA TTTGAGATGA
 241  GATAATCTCC TTCCTGACTT AAATTTTGTG CGTGCGTGCG AGAGAAACAT CTGAATCGAG
 301  AGGGACTTAA TGAGAAAAGC TTGGTCCCCT CCTTACCAAC TGTAGCACCT TGAAGCCCTG
 361  CAAGTTTTCC AACATACAAG GAGGAGATCA AATCCACTTG CCTTTGAGAA CTAGACCTCA
 421  GGGAGGAGGC TTCCGGCTCA GCACCAGAAC GAGTCTTCTA AGTTCTGTCT CTGAAGTTTG
 481  TGATGTCTTC AGTAACAAAG CTTACCCTCC AGTGTTTGGA GATTAAAGAT GAAAACCAGC
 541  TGTTGATTGT GAGAAAATGC ATCCAGGTTC ATAAATCCGG ACTCCAAGTG TAGTGACCAA
 601  AGTGGAGGTG CTGTGGAGTG TAATCATCAG AAGACCAGAT GGGCCTCTTA TATTCTCATC
 661  TGCATCAACC CCCCCCCCCC TTACTTCTAG CCTTACCACA TTGAGGATTT TATATAGTCA
 721  TTCATTTGTT TAGACAGGCT CTCACAATAC AGCCTAGGTT GTCCAAAGCT CACTATATAT
 781  CCCAGCTAAC AGTGAACTAC TCACACTACC CCTGCCTCAG CCTTCTGGGT GCTAGGATTA
 841  GAGCCATTAA GCCACAACCC ATGACAACTT CAAATAATTT TTCCAAATCC ATTTCCTCAT
 901  CTCTGAGGTG AGGGGCTGAT GCTAAAGCAT AAATCATAAG GTGAGTTTAG AAAAGGAGAT
 961  GGCGAGACCC ATAGCAAATG CTATCAAAAG GTGGACAGCA TCCACCATGG GAGTTTGGGA
1021  GTAAACTGAT TTCATGGGTC ACAGATGATG AAGAGGATAA TGGAGAATTT TTGTTGTGGG
1081  AGGAAGTCAT GTGGTTACAT GTGGAAGCCA TGAACAGGTG TACAGGGTAC CAATACAAAA
1141  TATTTCCTGT TGTGGATCAT GATCAATAAA GTTAGGTCAG CATTATGGTC TATTACTTTT
1201  GCCCACTTAT CATTTTGTTT GATTTTTTTC TCATGGTTTA GAATGTATCT CATTT
```

Supplementary Figure S2: Full-length cDNA of lncRNA-AK015487. (**A**) Nucleotide sequences of the full-length cDNA of lncRNA-AK015487 using 5′ and 3′ RACE analysis.

Supplementary Table S1: Predicted TFs for up-regulated mRNAs in HBx-transgenic mouse liver

TF name	Random LncRNA		TF name	2-folder changed lncRNA		TF name	4-folder changed lncRNA	
	count	ratio		count	ratio		count	ratio
SOX17	60	0.6	Evi1	872	0.77305	Evi1	117	0.769737
Sox5	54	0.54	MEF-2	763	0.676418	SOX17	107	0.703947
IRF	53	0.53	IRF-1	750	0.664894	Sox5	104	0.684211
S8	53	0.53	SOX17	749	0.664007	IRF-1	103	0.677632
IRF-1	50	0.5	IRF	649	0.575355	MEF-2	102	0.671053
myogenin	49	0.49	Sox5	637	0.564716	POU3F2	81	0.532895
Elf-1	45	0.45	POU3F2	635	0.562943	myogenin	80	0.526316
POU3F2	44	0.44	Pax-6	615	0.545213	IRF	78	0.513158
C/EBPgamma	44	0.44	S8	596	0.528369	S8	78	0.513158
ICSBP	44	0.44	E2A	540	0.478723	Pax-6	77	0.506579
Evi1	39	0.39	myogenin	536	0.475177	MAF	77	0.506579
MEF-2	38	0.38	Elf-1	525	0.465426	1-Oct	77	0.506579
Pax-6	38	0.38	PPARalpha:RXRalpha	500	0.443262	Nkx2-5	75	0.493421
FOX factors	38	0.38	T	497	0.440603	E2A	72	0.473684
Pax	38	0.38	1-Oct	492	0.43617	T	70	0.460526
PPAR direct repeat 1	38	0.38	MAF	492	0.43617	PPARalpha:RXRalpha	70	0.460526
KROX	38	0.38	Nkx2-5	485	0.429965	Elf-1	67	0.440789
T	37	0.37	C/EBPgamma	474	0.420213	FOX factors	65	0.427632
MAF	37	0.37	POU6F1	451	0.399823	Pax	64	0.421053
POU6F1	37	0.37	IRF-2	450	0.398936	POU6F1	62	0.407895
PPAR, HNF-4, COUP, RAR	37	0.37	FOX factors	446	0.39539	MTF-1	60	0.394737
POU2F2	34	0.34	Pax	446	0.39539	ICSBP	59	0.388158
LEF1, TCF1	34	0.34	FOXO1	437	0.387411	Spz1	59	0.388158
E2A	33	0.33	ICSBP	429	0.380319	PU.1	58	0.381579
1-Oct	32	0.32	Nkx2-2	427	0.378546	Nkx2-2	57	0.375
PU.1	32	0.32	Spz1	410	0.363475	IRF-2	56	0.368421
HOXA9	32	0.32	PU.1	407	0.360816	FOXO1	54	0.355263
IRF-2	31	0.31	MyoD	384	0.340426	FOXO4	54	0.355263
FOXO1	31	0.31	Sp1	376	0.333333	C/EBPgamma	52	0.342105
MyoD	31	0.31	Alx-4	374	0.33156	MyoD	52	0.342105
Pax5	31	0.31	Cart-1	374	0.33156	C/EBPbeta	52	0.342105
Egr	31	0.31	POU2F2	374	0.33156	Alx-4	51	0.335526
RXR-alpha	31	0.31	MTF-1	373	0.330674	Cart-1	51	0.335526
Alx-4	30	0.3	C/EBPbeta	366	0.324468	Sp1	51	0.335526
Cart-1	30	0.3	FOXO4	358	0.317376	LEF1, TCF1	50	0.328947
FOXJ1	30	0.3	GCNF	356	0.315603	HSF	49	0.322368
PPARalpha:RXRalpha	29	0.29	Pax5	345	0.305851	PPAR, HNF-4, COUP, RAR	48	0.315789
Nkx2-5	29	0.29	PPAR, HNF-4, COUP, RAR	340	0.301418	GCNF	48	0.315789
MTF-1	29	0.29	PPAR direct repeat 1	336	0.297872	POU2F2	47	0.309211

HOXA5 (Hox-1.3)	29	0.29	alpha-CP1	333	0.295213	alpha-CP1	47	0.309211
ERR alpha	29	0.29	LEF1, TCF1	331	0.29344	NF-kappaB	47	0.309211
Sp1	28	0.28	NF-kappaB	329	0.291667	FOXJ1	46	0.302632
VDR, CAR, PXR	28	0.28	HSF	324	0.287234	PPAR direct repeat 1	45	0.296053
ARNT	28	0.28	Ets	323	0.286348	HOXA5 (Hox-1.3)	44	0.289474
STATx	28	0.28	KROX	320	0.283688	ER	44	0.289474
Nkx2-2	27	0.27	SRF	316	0.280142	Egr	43	0.282895
alpha-CP1	27	0.27	NF-Y	315	0.279255	STAT5B (homodimer)	43	0.282895
Octamer	27	0.27	PPARG	314	0.278369	HNF1	43	0.282895
NF-AT	27	0.27	FOXJ1	307	0.272163	KROX	42	0.276316
Spz1	26	0.26	Lhx3	304	0.269504	p53 decamer	42	0.276316
FOXO4	26	0.26	Egr	301	0.266844	SRF	42	0.276316
GCNF	26	0.26	HOXA5 (Hox-1.3)	288	0.255319	NF-AT	41	0.269737
HSF	26	0.26	PPAR-gamma1	288	0.255319	Ik-3	41	0.269737
PPAR-gamma1	26	0.26	Octamer	286	0.253546	NF-Y	41	0.269737
NF-E2	26	0.26	PEBP	284	0.251773	Pax5	40	0.263158
TTF1 (Nkx2-1)	26	0.26	NF-E2	279	0.24734	VDR, CAR, PXR	40	0.263158
p53 decamer	26	0.26	STAT5B (homodimer)	279	0.24734	myogenin / NF-1	40	0.263158
Ets	25	0.25	SF-1	278	0.246454	PPARG	40	0.263158
STAT5B (homodimer)	25	0.25	myogenin / NF-1	275	0.243794	PPAR-gamma1	39	0.256579
STAT5A (homotetramer)	25	0.25	ER	266	0.235816	STAT5A (homodimer)	39	0.256579
Ikaros	25	0.25	HNF1	264	0.234043	ARNT	38	0.25
STAT5A (homodimer)	24	0.24	RXR-alpha	260	0.230496	Ets	38	0.25
PEBP	23	0.23	COMP1	259	0.22961	PEBP	38	0.25
COMP1	23	0.23	Crx	255	0.226064	p53	38	0.25
PR, GR	23	0.23	NF-AT	254	0.225177	COMP1	37	0.243421
C/EBPbeta	22	0.22	T3R	254	0.225177	RFX1	37	0.243421
SRF	22	0.22	TTF1 (Nkx2-1)	253	0.224291	RXR-alpha	36	0.236842
SF-1	22	0.22	VDR, CAR, PXR	251	0.222518	SF-1	36	0.236842
T3R	22	0.22	ERR alpha	248	0.219858	NF-E2 p45	36	0.236842
NF-E2 p45	22	0.22	HOXA9	248	0.219858	AML	36	0.236842
FOXF1	22	0.22	p53 decamer	248	0.219858	Octamer	34	0.223684
AML	21	0.21	C/EBPdelta	246	0.218085	T3R	34	0.223684
Lmo2 complex	21	0.21	NF-E2 p45	245	0.217199	FOXF1	34	0.223684
p53	20	0.2	STAT5A (homodimer)	242	0.214539	Lhx3	34	0.223684
NF-kappaB	19	0.19	Ik-3	240	0.212766	C/EBPdelta	34	0.223684
myogenin / NF-1	19	0.19	Lhx3a	237	0.210106	USF	34	0.223684
POU2F2 (Oct-2.1)	19	0.19	STAT5A (homotetramer)	234	0.207447	Lhx3a	34	0.223684
POU2F2 (Oct-2.3)	19	0.19	AP-1	230	0.203901	TTF1 (Nkx2-1)	33	0.217105
POU2F2 (Oct-2.4)	19	0.19	AhR:Arnt	228	0.202128	Crx	33	0.217105
POU2F2 (Oct-2.6)	19	0.19	POU2F2 (Oct-2.1)	227	0.201241	NF-E2	32	0.210526
PXR, CAR, LXR, FXR	19	0.19	POU2F2 (Oct-2.3)	227	0.201241	Ikaros	32	0.210526
TBP	19	0.19	POU2F2 (Oct-2.4)	227	0.201241	SF1	32	0.210526
POU6F1 (c2)	19	0.19	POU2F2 (Oct-2.6)	227	0.201241	ERR alpha	31	0.203947
Ik-1	19	0.19	FOXF1	223	0.197695	STATx	31	0.203947

Ik-2	19	0.19	AML	220	0.195035	STAT5A (homotetramer)	31	0.203947
PPARG	18	0.18	Tax/CREB	218	0.193262	POU2F2 (Oct-2.1)	30	0.197368
Lhx3	18	0.18	USF	218	0.193262	POU2F2 (Oct-2.3)	30	0.197368
ER	18	0.18	LXR	216	0.191489	POU2F2 (Oct-2.4)	30	0.197368
C/EBPdelta	18	0.18	RFX1	215	0.190603	POU2F2 (Oct-2.6)	30	0.197368
USF	18	0.18	p53	214	0.189716	LXR	30	0.197368
Ik-3	17	0.17	Ikaros	213	0.18883	HOXA9	29	0.190789
RFX1	17	0.17	Lmo2 complex	213	0.18883	Ik-1	29	0.190789
SF1	17	0.17	ARNT	212	0.187943	Ik-2	29	0.190789
Hmx3	17	0.17	PXR, CAR, LXR, FXR	210	0.18617	NF-kappaB (p65)	29	0.190789
LXR direct repeat 4	17	0.17	TBP	204	0.180851	PPAR-alpha:RXR-alpha	29	0.190789
EBF1	17	0.17	STATx	202	0.179078	PXR, CAR, LXR, FXR	28	0.184211
Stra13	17	0.17	RFX1 (EF-C)	195	0.172872	LXR-beta:RXR-alpha	28	0.184211
NF-Y	16	0.16	SF1	194	0.171986	Lmo2 complex	27	0.177632
HNF1	16	0.16	POU6F1 (c2)	190	0.16844	Stra13	27	0.177632
Crx	16	0.16	Hmx3	181	0.160461	AhR:Arnt	27	0.177632
AhR:Arnt	16	0.16	LXR-beta:RXR-alpha	181	0.160461	RFX1 (EF-C)	26	0.171053
LXR-beta:RXR-alpha	16	0.16	Pax-8	181	0.160461	Pax-8	26	0.171053
Pax-4a	16	0.16	RFX2	181	0.160461	RFX2	26	0.171053
RFX1 (EF-C)	15	0.15	RFX3	181	0.160461	RFX3	26	0.171053
HSF1	15	0.15	Ik-1	180	0.159574	TBP	25	0.164474
HNF-1alpha	15	0.15	Ik-2	180	0.159574	ER-alpha	25	0.164474
NF-TNF	15	0.15	NF-kappaB (p65)	175	0.155142	RelA-p65	24	0.157895
Lhx3a	14	0.14	PR, GR	175	0.155142	AP-1	24	0.157895
NF-kappaB (p65)	14	0.14	PPAR-alpha:RXR-alpha	173	0.153369	CREB	24	0.157895
ER-alpha	14	0.14	ER-alpha	170	0.150709	Tax/CREB	23	0.151316
Zic1	14	0.14	PXR-1:RXR-alpha	170	0.150709	Hmx3 (Nkx5-1)	23	0.151316
STAT	13	0.13	STAT	170	0.150709	PR, GR	22	0.144737
RelA-p65	13	0.13	Zic1	167	0.14805	EBF1	22	0.144737
PPAR-gamma:RXR-alpha	13	0.13	E2F	164	0.14539	Zic1	22	0.144737
GKLF	13	0.13	POU5F1	163	0.144504	Brachyury	22	0.144737
LXR	12	0.12	GCM	162	0.143617	Hmx3	21	0.138158
Pax-8	12	0.12	LXR direct repeat 4	162	0.143617	GCM	21	0.138158
RFX2	12	0.12	EBF1	159	0.140957	PXR-1:RXR-alpha	21	0.138158
RFX3	12	0.12	HSF1	156	0.138298	POU5F1	21	0.138158
GCM	12	0.12	Stra13	153	0.135638	LXR direct repeat 4	20	0.131579
Brachyury	12	0.12	Hmx3 (Nkx5-1)	151	0.133865	HNF-1alpha	20	0.131579
NF-kappaB1	12	0.12	ERR1	148	0.131206	HSF1	19	0.125
PPAR-alpha	12	0.12	CAR:RXR-alpha	146	0.129433	ERR1	19	0.125
PXR-1:RXR-alpha	11	0.11	HNF-1alpha	146	0.129433	NF-TNF	18	0.118421
c-Ets-1(p54)	11	0.11	CREB	145	0.128546	NF-kappaB1	18	0.118421
PPAR-alpha:RXR-alpha	10	0.1	Brachyury	135	0.119681	c-Ets-1(p54)	18	0.118421

ERR1	10	0.1	C/EBP	132	0.117021	E2F	18	0.118421
Zic2	10	0.1	NF-TNF	126	0.111702	POU6F1 (c2)	17	0.111842
LXR-alpha:RXR-alpha	10	0.1	c-Ets-1(p54)	125	0.110816	C/EBP	17	0.111842
RFX	10	0.1	RelA-p65	125	0.110816	PPAR-gamma:RXR-alpha	16	0.105263
AP-1	9	0.09	PPAR-gamma:RXR-alpha	123	0.109043	STAT	15	0.098684
Tax/CREB	9	0.09	NF-kappaB1	122	0.108156	PPAR-alpha	15	0.098684
E2F	9	0.09	Zic2	121	0.10727	c-Rel	15	0.098684
POU5F1	9	0.09	Pax-4a	110	0.097518	CAR:RXR-alpha	13	0.085526
CREB	9	0.09	PPAR-alpha	105	0.093085	Pax-4a	12	0.078947
Ncx	9	0.09	LXR-alpha:RXR-alpha	100	0.088652	LXR-alpha:RXR-alpha	12	0.078947
CAR:RXR-alpha	8	0.08	c-Rel	93	0.082447	Pax-1	12	0.078947
Pax-1	8	0.08	Pax-1	92	0.08156	NF-kappaB(-like)	12	0.078947
STAT3	8	0.08	CRE-BP2	89	0.078901	GKLF	11	0.072368
AhR	8	0.08	STAT3	89	0.078901	RFX	11	0.072368
Egr-1	8	0.08	Zic3	87	0.077128	Zic2	10	0.065789
C/EBP	7	0.07	NF-kappaB(-like)	86	0.076241	AhR	10	0.065789
c-Rel	7	0.07	GKLF	78	0.069149	SRF-L	10	0.065789
NF-kappaB(-like)	7	0.07	AhR	72	0.06383	STAT3	9	0.059211
Hmx3 (Nkx5-1)	6	0.06	Ncx	72	0.06383	CRE-BP2	9	0.059211
CRE-BP2	6	0.06	RFX	72	0.06383	Ncx	8	0.052632
Zic3	6	0.06	SRF-L	64	0.056738	Zic3	8	0.052632
SRF-L	6	0.06	E2F-1	54	0.047872	RXR-beta	8	0.052632
CREB, ATF	6	0.06	Egr-1	54	0.047872	E2F-1	7	0.046053
E2F-1	5	0.05	RXR-beta	53	0.046986	GCMa	7	0.046053
RXR-beta	5	0.05	STAT1alpha	50	0.044326	GCMb	7	0.046053
STAT1alpha	5	0.05	HOXA5	45	0.039894	CREB, ATF	6	0.039474
MEF-2DAB	5	0.05	MEF-2DAB	45	0.039894	STAT1alpha	6	0.039474
EBF	5	0.05	GCMa	44	0.039007	MEF-2DAB	6	0.039474
HOXA5	3	0.03	GCMb	44	0.039007	EBF	6	0.039474
PEBP2alphaB1	3	0.03	PEBP2alphaB1	42	0.037234	Pax-3	6	0.039474
Pax-3	3	0.03	CREB, ATF	40	0.035461	c-Jun	6	0.039474
Meis-1b	2	0.02	c-Jun	39	0.034574	PEBP2alphaB1	5	0.032895
GCMa	1	0.01	EBF	39	0.034574	STAT5A	5	0.032895
GCMb	1	0.01	Pax-3	32	0.028369	Egr-1	3	0.019737
c-Jun	1	0.01	Lyf-1	28	0.024823	HOXA5	2	0.013158
Lyf-1	1	0.01	STAT5A	24	0.021277	Meis-1b	2	0.013158
STAT5A	1	0.01	Meis-1b	22	0.019504	Lyf-1	2	0.013158

Supplementary Table S2: Predicted TFs for up-regulated lncRNAs in HBx-transgenic mouse liver

TF name	Random mRNA		TF name	2-folder changed mRNA		TF name	4-folder changed mRNA	
	count	Ratio		count	Ratio		count	Ratio
SOX17	63	0.63	Evi1	1528	0.697717	Evi1	256	0.707182
Sox5	59	0.59	MEF-2	1316	0.600913	MEF-2	216	0.596685
IRF	55	0.55	IRF-1	1273	0.581279	IRF-1	206	0.569061
S8	50	0.5	SOX17	1265	0.577626	SOX17	192	0.530387
IRF-1	50	0.5	IRF	1151	0.525571	Sox5	189	0.522099
myogenin	46	0.46	Sp1	1086	0.49589	IRF	180	0.497238
Elf-1	46	0.46	Sox5	1081	0.493607	Sp1	178	0.491713
C/EBPgamma	45	0.45	Pax-6	1078	0.492237	E2A	173	0.477901
ICSBP	45	0.45	POU3F2	1029	0.469863	myogenin	169	0.466851
POU3F2	43	0.43	E2A	1006	0.459361	PPARalpha:RXRalpha	165	0.455801
Evi1	41	0.41	myogenin	1002	0.457534	Pax-6	164	0.453039
Pax	40	0.4	S8	997	0.455251	S8	163	0.450276
Pax-6	37	0.37	Elf-1	980	0.447489	Elf-1	161	0.444751
FOX factors	37	0.37	MAF	940	0.429224	POU3F2	159	0.439227
KROX	37	0.37	1-Oct	940	0.429224	Pax	150	0.414365
MEF-2	36	0.36	PPARalpha:RXRalpha	937	0.427854	T	140	0.38674
PPAR direct repeat 1	36	0.36	T	868	0.396347	MAF	138	0.381215
T	36	0.36	KROX	835	0.381279	1-Oct	138	0.381215
MAF	34	0.34	Pax	832	0.379909	FOX factors	134	0.370166
POU6F1	34	0.34	PU.1	806	0.368037	Nkx2-2	133	0.367403
PPAR, HNF-4, COUP, RAR	34	0.34	FOX factors	793	0.3621	Spz1	132	0.364641
POU2F2	33	0.33	IRF-2	793	0.3621	NF-Y	132	0.364641
LEF1, TCF1	33	0.33	Nkx2-5	783	0.357534	IRF-2	130	0.359116
E2A	33	0.33	C/EBPgamma	775	0.353881	C/EBPgamma	129	0.356354
1-Oct	32	0.32	Nkx2-2	756	0.345205	KROX	128	0.353591
PU.1	32	0.32	Spz1	744	0.339726	alpha-CP1	127	0.350829
HOXA9	31	0.31	ICSBP	736	0.336073	FOXO1	123	0.339779
MyoD	31	0.31	MyoD	733	0.334703	POU6F1	123	0.339779
Egr	31	0.31	Egr	728	0.33242	Nkx2-5	121	0.334254
IRF-2	30	0.3	FOXO1	722	0.32968	C/EBPbeta	121	0.334254
FOXO1	30	0.3	POU6F1	720	0.328767	MyoD	120	0.331492
Pax5	30	0.3	C/EBPbeta	711	0.324658	PU.1	118	0.325967
RXR-alpha	29	0.29	Alx-4	677	0.309132	Egr	117	0.323204
Alx-4	29	0.29	Cart-1	677	0.309132	NF-kappaB	116	0.320442
Cart-1	29	0.29	MTF-1	672	0.306849	ICSBP	114	0.314917
FOXJ1	29	0.29	NF-Y	662	0.302283	Alx-4	113	0.312155
PPARalpha:RXRalpha	29	0.29	alpha-CP1	649	0.296347	Cart-1	113	0.312155
Nkx2-5	28	0.28	Pax5	642	0.293151	MTF-1	109	0.301105
MTF-1	28	0.28	NF-kappaB	634	0.289498	SRF	104	0.287293
ERR alpha	28	0.28	PPAR direct repeat 1	606	0.276712	GCNF	102	0.281768
HOXA5 (Hox-1.3)	27	0.27	Ets	605	0.276256	Pax5	99	0.273481
Sp1	27	0.27	FOXO4	583	0.26621	VDR, CAR, PXR	98	0.270718
VDR, CAR, PXR	27	0.27	SRF	578	0.263927	POU2F2	97	0.267956

ARNT	27	0.27	GCNF	577	0.26347	HSF	97	0.267956
STATx	27	0.27	POU2F2	575	0.262557	PPAR direct repeat 1	95	0.262431
alpha-CP1	27	0.27	PPAR, HNF-4, COUP, RAR	572	0.261187	FOXO4	95	0.262431
NF-AT	27	0.27	HSF	566	0.258447	Ets	94	0.259669
Nkx2-2	26	0.26	PPARG	561	0.256164	NF-AT	93	0.256906
Octamer	26	0.26	STAT5B (homodimer)	559	0.255251	myogenin / NF-1	92	0.254144
PPAR-gamma1	25	0.25	myogenin / NF-1	546	0.249315	HOXA5 (Hox-1.3)	92	0.254144
Spz1	24	0.24	VDR, CAR, PXR	536	0.244749	HNF1	92	0.254144
FOXO4	24	0.24	FOXJ1	533	0.243379	PPARG	91	0.251381
HSF	24	0.24	LEF1, TCF1	530	0.242009	STAT5B (homodimer)	91	0.251381
NF-E2	24	0.24	HOXA5 (Hox-1.3)	527	0.240639	PPAR, HNF-4, COUP, RAR	90	0.248619
GCNF	23	0.23	PPAR-gamma1	524	0.239269	NF-E2	89	0.245856
TTF1 (Nkx2-1)	23	0.23	SF-1	523	0.238813	FOXJ1	88	0.243094
p53 decamer	23	0.23	NF-E2	517	0.236073	PPAR-gamma1	86	0.237569
Ets	23	0.23	STAT5A (homodimer)	493	0.225114	Ik-3	86	0.237569
STAT5B (homodimer)	23	0.23	NF-AT	475	0.216895	STAT5A (homodimer)	84	0.232044
STAT5A (homotetramer)	23	0.23	ER	469	0.214155	STAT5A (homotetramer)	81	0.223757
Ikaros	23	0.23	HNF1	469	0.214155	LEF1, TCF1	80	0.220994
STAT5A (homodimer)	22	0.22	ERR alpha	468	0.213699	SF-1	80	0.220994
PEBP	22	0.22	Octamer	463	0.211416	COMP1	79	0.218232
COMP1	22	0.22	p53 decamer	459	0.209589	ER	77	0.212707
PR, GR	21	0.21	AhR:Arnt	459	0.209589	PEBP	76	0.209945
C/EBPbeta	21	0.21	PEBP	457	0.208676	Octamer	75	0.207182
SF-1	21	0.21	RXR-alpha	456	0.208219	TTF1 (Nkx2-1)	75	0.207182
T3R	21	0.21	TTF1 (Nkx2-1)	456	0.208219	Lhx3	75	0.207182
SRF	20	0.2	STAT5A (homotetramer)	456	0.208219	Crx	74	0.20442
NF-E2 p45	20	0.2	Tax/CREB	455	0.207763	Pax-8	73	0.201657
FOXF1	20	0.2	Ik-3	452	0.206393	RXR-alpha	71	0.196133
AML	20	0.2	Lmo2 complex	449	0.205023	Tax/CREB	71	0.196133
Lmo2 complex	20	0.2	p53	447	0.20411	AhR:Arnt	69	0.190608
p53	19	0.19	Crx	443	0.202283	HOXA9	69	0.190608
NF-kappaB	19	0.19	Lhx3	441	0.20137	ERR alpha	68	0.187845
myogenin / NF-1	18	0.18	COMP1	439	0.200457	p53 decamer	68	0.187845
PXR, CAR, LXR, FXR	18	0.18	NF-E2 p45	432	0.19726	p53	68	0.187845
POU6F1 (c2)	18	0.18	ARNT	430	0.196347	NF-E2 p45	68	0.187845
Ik-1	18	0.18	Ikaros	428	0.195434	STATx	67	0.185083
POU2F2 (Oct-2.1)	17	0.17	STATx	406	0.185388	PXR, CAR, LXR, FXR	67	0.185083
POU2F2 (Oct-2.3)	17	0.17	T3R	401	0.183105	Ik-1	67	0.185083
TBP	17	0.17	PXR, CAR, LXR, FXR	397	0.181279	Ik-2	67	0.185083
Ik-2	17	0.17	C/EBPdelta	392	0.178995	ER-alpha	67	0.185083
POU2F2 (Oct-2.4)	16	0.16	USF	387	0.176712	Lmo2 complex	65	0.179558

POU2F2 (Oct-2.6)	16	0.16	HOXA9	384	0.175342	GCM	65	0.179558
PPARG	16	0.16	RFX1	383	0.174886	Ikaros	64	0.176796
Lhx3	16	0.16	AP-1	374	0.170776	FOXF1	63	0.174033
ER	16	0.16	Ik-1	371	0.169406	Zic1	63	0.174033
USF	16	0.16	Ik-2	371	0.169406	USF	62	0.171271
C/EBPdelta	15	0.15	AML	364	0.16621	AML	61	0.168508
Ik-3	15	0.15	LXR	362	0.165297	TBP	61	0.168508
RFX1	15	0.15	Pax-8	362	0.165297	LXR direct repeat 4	61	0.168508
SF1	15	0.15	STAT	350	0.159817	T3R	60	0.165746
Hmx3	15	0.15	TBP	347	0.158447	RFX1	60	0.165746
EBF1	15	0.15	Lhx3a	344	0.157078	AP-1	60	0.165746
Stra13	15	0.15	POU2F2 (Oct-2.1)	341	0.155708	NF-kappaB (p65)	60	0.165746
LXR direct repeat 4	14	0.14	POU2F2 (Oct-2.3)	341	0.155708	ARNT	59	0.162983
NF-Y	14	0.14	POU2F2 (Oct-2.4)	341	0.155708	LXR	59	0.162983
HNF1	14	0.14	POU2F2 (Oct-2.6)	341	0.155708	Lhx3a	59	0.162983
Crx	14	0.14	E2F	340	0.155251	POU6F1 (c2)	59	0.162983
AhR:Arnt	14	0.14	GCM	337	0.153881	SF1	57	0.157459
LXR-beta:RXR-alpha	14	0.14	CREB	337	0.153881	STAT	56	0.154696
Pax-4a	14	0.14	FOXF1	333	0.152055	C/EBPdelta	54	0.149171
RFX1 (EF-C)	14	0.14	Zic1	333	0.152055	EBF1	54	0.149171
HSF1	13	0.13	SF1	325	0.148402	POU2F2 (Oct-2.1)	53	0.146409
HNF-1alpha	13	0.13	LXR-beta:RXR-alpha	315	0.143836	POU2F2 (Oct-2.3)	53	0.146409
NF-TNF	13	0.13	PR, GR	312	0.142466	POU2F2 (Oct-2.4)	53	0.146409
NF-kappaB (p65)	13	0.13	RFX1 (EF-C)	310	0.141553	POU2F2 (Oct-2.6)	53	0.146409
Lhx3a	12	0.12	PPAR-alpha:RXR-alpha	308	0.140639	NF-kappaB1	52	0.143646
ER-alpha	12	0.12	Hmx3	304	0.138813	PXR-1:RXR-alpha	51	0.140884
Zic1	12	0.12	NF-kappaB (p65)	300	0.136986	LXR-beta:RXR-alpha	50	0.138122
STAT	12	0.12	PXR-1:RXR-alpha	299	0.13653	PPAR-alpha:RXR-alpha	50	0.138122
RelA-p65	12	0.12	POU6F1 (c2)	291	0.132877	RFX2	47	0.129834
PPAR-gamma:RXR-alpha	11	0.11	ER-alpha	290	0.13242	RFX3	47	0.129834
GKLF	11	0.11	RFX2	285	0.130137	CREB	46	0.127072
LXR	11	0.11	RFX3	285	0.130137	RFX1 (EF-C)	46	0.127072
Pax-8	11	0.11	EBF1	282	0.128767	E2F	45	0.124309
RFX2	11	0.11	Stra13	282	0.128767	Hmx3	45	0.124309
RFX3	11	0.11	LXR direct repeat 4	280	0.127854	NF-TNF	45	0.124309
GCM	11	0.11	NF-kappaB1	277	0.126484	RelA-p65	45	0.124309
Brachyury	11	0.11	POU5F1	271	0.123744	C/EBP	45	0.124309
NF-kappaB1	11	0.11	NF-TNF	266	0.121461	PR, GR	43	0.118785
PPAR-alpha	11	0.11	HSF1	261	0.119178	HSF1	43	0.118785
PXR-1:RXR-alpha	10	0.1	c-Ets-1(p54)	252	0.115068	ERR1	41	0.11326
c-Ets-1(p54)	10	0.1	RelA-p65	251	0.114612	HNF-1alpha	40	0.110497
PPAR-alpha:RXR-alpha	10	0.1	ERR1	245	0.111872	Zic2	38	0.104972
ERR1	10	0.1	Hmx3 (Nkx5-1)	245	0.111872	CAR:RXR-alpha	38	0.104972

Zic2	10	0.1	C/EBP	241	0.110046	c-Rel	37	0.10221
LXR-alpha:RXR-alpha	10	0.1	HNF-1alpha	236	0.107763	POU5F1	36	0.099448
RFX	10	0.1	Brachyury	234	0.106849	Hmx3 (Nkx5-1)	36	0.099448
AP-1	10	0.1	Zic2	227	0.103653	NF-kappaB(-like)	36	0.099448
Tax/CREB	10	0.1	PPAR-gamma:RXR-alpha	224	0.102283	PPAR-alpha	35	0.096685
E2F	10	0.1	CAR:RXR-alpha	213	0.09726	Stra13	34	0.093923
POU5F1	9	0.09	c-Rel	210	0.09589	c-Ets-1(p54)	33	0.09116
CREB	9	0.09	STAT3	206	0.094064	Pax-1	33	0.09116
Ncx	8	0.08	GKLF	204	0.093151	Brachyury	32	0.088398
CAR:RXR-alpha	8	0.08	PPAR-alpha	204	0.093151	PPAR-gamma:RXR-alpha	31	0.085635
Pax-1	8	0.08	Pax-4a	201	0.091781	GKLF	29	0.08011
STAT3	8	0.08	NF-kappaB(-like)	187	0.085388	STAT3	28	0.077348
AhR	7	0.07	Pax-1	180	0.082192	CRE-BP2	28	0.077348
Egr-1	7	0.07	LXR-alpha:RXR-alpha	169	0.077169	Zic3	28	0.077348
c-Rel	7	0.07	Egr-1	169	0.077169	STAT1alpha	28	0.077348
NF-kappaB(-like)	7	0.07	AhR	167	0.076256	Pax-4a	27	0.074586
C/EBP	6	0.06	CRE-BP2	166	0.075799	Egr-1	26	0.071823
Hmx3 (Nkx5-1)	6	0.06	Zic3	165	0.075342	LXR-alpha:RXR-alpha	23	0.063536
CRE-BP2	6	0.06	E2F-1	143	0.065297	RFX	22	0.060773
Zic3	6	0.06	SRF-L	131	0.059817	AhR	21	0.058011
SRF-L	6	0.06	RFX	128	0.058447	SRF-L	21	0.058011
CREB, ATF	5	0.05	STAT1alpha	125	0.057078	E2F-1	18	0.049724
E2F-1	5	0.05	Ncx	107	0.048858	EBF	18	0.049724
STAT1alpha	5	0.05	CREB, ATF	106	0.048402	HOXA5	18	0.049724
RXR-beta	4	0.04	GCMa	94	0.042922	GCMa	16	0.044199
MEF-2DAB	4	0.04	GCMb	94	0.042922	GCMb	16	0.044199
EBF	4	0.04	EBF	82	0.037443	c-Jun	16	0.044199
HOXA5	4	0.04	HOXA5	80	0.03653	Ncx	14	0.038674
PEBP2alphaB1	4	0.04	RXR-beta	79	0.036073	MEF-2DAB	14	0.038674
Pax-3	4	0.04	c-Jun	78	0.035616	PEBP2alphaB1	13	0.035912
Meis-1b	3	0.03	MEF-2DAB	77	0.03516	CREB, ATF	12	0.033149
GCMa	3	0.03	PEBP2alphaB1	77	0.03516	RXR-beta	9	0.024862
GCMb	3	0.03	STAT5A	60	0.027397	STAT5A	6	0.016575
c-Jun	1	0.01	Pax-3	57	0.026027	Lyf-1	4	0.01105
Lyf-1	1	0.01	Lyf-1	41	0.018721	Meis-1b	4	0.01105
STAT5A	1	0.01	Meis-1b	24	0.010959	Pax-3	2	0.005525

Supplementary Table S3A: Randomly selected lncRNAs for transcription factor binding site prediction

GenbankAccession	chr	strand	start	end
AK036360	chr9	−	112080591	112082978
AK015287	chr5	−	106793579	106795868
AK015305	chr19	−	58948419	58949628
AK015266	chr10	−	44450574	44458706
AK015840	chr5	+	41672474	41674139
AK080243	chr11	+	96668514	96669461
AK036557	chr7	+	142729506	142732878
AK015623	chr10	+	92544041	92544641
AK036231	chr11	+	69485178	69488170
AK036508	chr6	+	92053631	92056101
AK015637	chr4	+	43013017	43013929
AK005763	chr19	+	41338601	41339520
AB046930	chr16	−	92644036	92644396
uc.320−	chr7	−	116346458	116346793
AK036205	chr12	+	30219769	30470670
AK015582	chr5	+	121953521	121954610
AK036433	chr2	+	12925991	12929227
AK015736	chr3	−	104314021	104315236
uc.312−	chr19	−	60281370	60281692
AK015307	chr1	+	92050202	92056037
AK048101	chr3	−	89573949	89577322
AK015815	chr4	+	44682201	44683769
uc.102+	chr2	+	72937887	72938225
AK015276	chr3	−	33765742	33796506
AK015295	chr17	+	15344565	15346353
AK015564	chr6	−	146724411	146725257
AK015779	chr5	−	39853803	39994807
AF093701	chrX	+	64203463	64203728
AK015727	chr11	−	80663882	80671802
AK015477	chr7	+	127040206	127041886
AK046926	chr17	+	10512791	10515471
AK015596	chr5	+	66342543	66343444
AK015846	chr11	−	33993042	33994126
AK015865	chr10	−	67425226	67436456
AK015764	chr6	−	149309857	149311132
AK015601	chr6	−	137094396	137095450
AK015835	chr13	−	50848761	50860617
AK015743	chr4	+	94223236	94223769
AF022856	chr1	+	62750278	62862462
AF061179	chr18	−	75746231	75747219
AK015707	chr7	+	38454817	38457247
AK015514	chr6	−	91962387	91966111
AF020312	chr11	+	69177518	69182908
AK015733	chr9	−	72366068	72379665
AK015352	chr2	−	74136996	74145774
AK015459	chr10	−	21038893	21061861

AK015508	chr9	−	115956784	115959984
AK015398	chr6	−	146794797	146795725
AK015390	chr6	−	29260771	29262223
AF019615	chr5	+	28495015	28495380
AK015453	chr17	+	86629825	86632038
AK047638	chr4	−	126010826	126015875
AK036235	chr6	−	97456069	97460255
AK015366	chr17	+	27462298	27463953
AK036483	chr9	+	76599242	76602513
AB056897	chr14	−	61893594	61893904
AK015531	chr1	+	37717566	37719492
AK015450	chr13	+	46031408	46032273
AK047090	chr5	+	135638124	135639740
AK015648	chr8	+	118109291	118119052
uc.319−	chr7	−	116334875	116335191
AK015693	chr9	−	74918196	74918961
AK015424	chr13	+	98750758	98751397
AK015487	chr10	+	95688840	95711342
uc.308−	chr19	−	45550666	45550943
AK015433	chr2	−	179385452	179386327
AK015387	chr2	−	96827174	96923718
AK015554	chr6	−	91380453	91390848
AB306984	chr17	+	40698455	40702573
AK015838	chr13	+	30706911	30716128
AK015344	chr10	+	75323087	75324443
uc.325−	chr2	+	105619687	105619922
AK015449	chr10	−	56156161	56157409
AK015695	chr15	+	69100962	69105148
AK015353	chr9	+	106543258	106545895
AK046990	chr17	+	47824558	47828032
AK015461	chr18	−	32823295	32996941
AK080301	chrX	+	35548338	35550220
AK015639	chr5	−	130619188	130620545
AK015618	chr7	+	140319933	140334140
AK048043	chr17	−	22493867	22498381
AK015443	chr4	+	141208038	141208577
AK047805	chr5	−	108550747	108552526
AK047380	chr6	+	50150160	50153118
AK036570	chr3	−	127039311	127201919
AK047418	chr5	+	125937155	125940739
AK036481	chr6	−	38255302	38257158
AF104996	chr6	−	69557273	69557545
AK015822	chr5	−	48394304	48396946
AK015539	chr8	+	62304445	62323518
AK036548	chr19	+	45519988	45523446
uc.310−	chr19	−	55551684	55551913
AK015613	chr10	−	119262259	119289446

AK015809	chr2	−	123949371	123955692
AK015332	chr1	+	190796397	190797618
AK036400	chr2	+	92324082	92369966
AK015451	chrX	−	20783706	20798791
AK080279	chr10	−	114820973	114822849
AK015605	chr3	+	94819546	94820992
AK047689	chr4	−	131468334	131471431

Supplementary Table S3B: Randomly selected mRNAs for transcription factor binding site prediction

gene	GenbankAccession	chr	stand	txstart	txend
Olfr1307	NM_001011787	chr2	−	111784672	111785611
BC055004	NM_001013773	chr5	+	138667131	138693103
Lrrc16b	NM_001024645	chr14	+	56109929	56127101
Cpa2	NM_001024698	chr6	+	30491641	30514473
Atad5	NM_001029856	chr11	+	79902901	79949293
Sybu	NM_001032727	chr15	−	44503402	44619609
AU021092	NM_001033220	chr16	−	5211911	5222392
Cgn	NM_001037711	chr3	−	94563991	94590437
Plekhm3	NM_001039493	chr1	−	64835694	65003398
Zhx1	NM_001042438	chr15	−	57878557	57908044
Nccrp1	NM_001081115	chr7	−	29328615	29332273
Alg6	NM_001081264	chr4	+	99382320	99430151
Duox1	NM_001099297	chr2	+	122141407	122173708
Samhd1	NM_001139520	chr2	−	156923264	156960958
Rbm43	NM_001141981	chr2	−	51779968	51790529
Rbm43	NM_001141982	chr2	−	51779968	51790164
Myo9b	NM_001142323	chr8	+	73796612	73884611
Alg12	NM_001142357	chr15	−	88635672	88649748
Vipar	NM_001142581	chr12	−	88579824	88607236
Arpp19	NM_001142655	chr9	+	74885420	74908120
Actb	NM_007393	chr5	−	143664794	143668403
E2f1	NM_007891	chr2	−	154385374	154395588
Fabp2	NM_007980	chr3	+	122598309	122602424
Gpi1	NM_008155	chr7	−	34986345	35015324
Gsta2	NM_008182	chr9	−	78178826	78194952
Gstm6	NM_008184	chr3	−	107741765	107746667
Il2	NM_008366	chr3	−	37019641	37024876
Lhx1	NM_008498	chr11	−	84332880	84339036
Ascl1	NM_008553	chr10	−	86953785	86956405
Strbp	NM_009261	chr2	−	37425387	37502805
Tcp10a	NM_009340	chr17	+	7529082	7549861
Tcp10a	NM_009340	chr17	+	13254056	13569627
Tgoln2	NM_009444	chr6	−	72560950	72566742
Zhx1	NM_009572	chr15	−	57878557	57908044
Apoe	NM_009696	chr7	−	20281592	20284515
Eci1	NM_010023	chr17	+	24563627	24576261
Gamt	NM_010255	chr10	−	79720895	79723713
Gstm3	NM_010359	chr3	−	107766613	107772092

H2−Q7	NM_010394	chr17	+	35576099	35580718
Pex11a	NM_011068	chr7	−	86882149	86887911
Ppara	NM_011144	chr15	+	85566205	85637281
Prdx2	NM_011563	chr8	+	87493546	87498212
Tuba1b	NM_011654	chr15	−	98761861	98764821
Tyr	NM_011661	chr7	−	94575914	94641921
Zfp54	NM_011760	chr17	+	21560190	21572348
Gnpda1	NM_011937	chr18	−	38487190	38498647
Anxa8	NM_013473	chr14	+	34899175	34913754
Hspb7	NM_013868	chr4	+	140976693	140981225
Nup50	NM_016714	chr15	+	84753857	84773393
Ostf1	NM_017375	chr19	−	18654853	18706303
Samhd1	NM_018851	chr2	−	156923264	156960958
Herpud2	NM_020586	chr9	−	24912573	24956280
Nop10	NM_025403	chr2	+	112102125	112103055
1110059E24Rik	NM_025423	chr19	−	21671802	21727281
Snrnp40	NM_025645	chr4	+	130037378	130067278
Snrnp27	NM_025665	chr6	−	86625162	86634485
Carhsp1	NM_025821	chr16	−	8658679	8672246
Rab5a	NM_025887	chr17	+	53618558	53647003
1110008F13Rik	NM_026124	chr2	+	156688857	156699298
Ankrd33b	NM_026153	chr15	−	31253709	31297514
Slc48a1	NM_026353	chr15	+	97614795	97623123
Snrnp48	NM_026382	chr13	+	38296807	38319532
Gar1	NM_026578	chr3	−	129527829	129534314
Gstm7	NM_026672	chr3	−	107729251	107734663
Gkn3	NM_026860	chr6	−	87333312	87338929
Gsdmd	NM_026960	chr15	+	75692768	75697834
2010001M09Rik	NM_027222	chr18	−	35806918	35809021
Hrct1	NM_027511	chr4	+	43740069	43740982
Fam57a	NM_027773	chr11	+	76015557	76021759
Alkbh4	NM_028070	chr5	+	136614650	136617484
Msl1	NM_028722	chr11	+	98657082	98669173
Tm9sf1	NM_028780	chr14	−	56254802	56262643
Uhrf1bp1l	NM_029166	chr10	+	89207735	89282614
Nop14	NM_029278	chr5	−	34981184	35002797
Snrnp35	NM_029532	chr5	+	124933163	124941131
Snx27	NM_029721	chr3	−	94301465	94386624
Wdyhv1	NM_029734	chr15	+	57972990	57990209
Snrnp25	NM_030093	chr11	+	32105414	32108995
Rbm43	NM_030243	chr2	−	51779968	51790529
Kcnn2	NM_080465	chr18	+	45719807	45845537
Sult1a1	NM_133670	chr7	−	133816383	133819871
Vipar	NM_134044	chr12	−	88579824	88605194
Gldc	NM_138595	chr19	−	30172936	30249908
Nop2	NM_138747	chr6	+	125081900	125094771
Ccdc21	NM_144527	chr4	−	133685772	133743000

Alg12	NM_145477	chr15	−	88635672	88649748
Sft2d2	NM_145512	chr1	−	167104471	167124564
Mmgt1	NM_146234	chrX	−	53838688	53851096
Olfr166	NM_147068	chr16	+	19486932	19487871
Nrn1	NM_153529	chr13	−	36817494	36826323
Dnajc11	NM_172704	chr4	+	151307828	151356068
St6gal2	NM_172829	chr17	+	55585014	55638524
Catsperb	NM_173023	chr12	+	102642882	102864219
Sh2d7	NM_173778	chr9	+	54386790	54392827
C730048C13Rik	NM_177002	chr19	−	8410112	8479595
Acaa2	NM_177470	chr18	+	74938865	74965861
Vangl1	NM_177545	chr3	−	101960618	102008616
Zfp507	NM_177739	chr7	−	36557368	36588008
Hyal3	NM_178020	chr9	+	107483626	107489690
Fbxl21	NM_178674	chr13	+	56623868	56639147

Supplementary Table S4: Oligonucleotide sequences used in this study

Gene	Name	Sequences
**q-PCR primers**		
AK015487	Sense	5′-CGGCTCAGCACCAGAACGA-3′
	Anti-sense	5′-CACTCCACAGCACCTCCACTT-3′
AK021106	Sense	5′-ACAGTCCTGGAAGAAATGATGA-3′
	Anti-sense	5′-AAGAACAAACCCGACCGTAT-3′
AK016494	Sense	5′-ACTTGGGACAAATGGAGTGAC-3′
	Anti-sense	5′-TCTGGAAGGCAGGTGTAGGA-3′
AK044545	Sense	5′-GCTGTGCGGAGCATCTAGG-3′
	Anti-sense	5′-TGCCAGGACAGGGAATCTACTT-3′
mβ-actin	Sense	5′-GCACCACACCTTCTACAATGAG-3′
	Anti-sense	5′-ACAGCCTGGATGGCTACGT-3′
hβ-actin	Sense	5′-ACACTGTGCCCATCTACGAGG -3′
	Anti-sense	5′-AGGGGCCGGACTCGTCATACT-3′
HBx	Sense	5′-TGCCTCATCTTCTTRTTGGTTCT-3′
	Anti-sense	5′-CCCCAAWACCAVATCATCCATATA-3′
mEvi1	Sense	5′-ATGGTGGAGGAGGACTTGC-3′
	Anti-sense	5′-GTGACATCTGGTGGCGAAT-3′
hEVI1	Sense	5′-GACCAAGTTTTTCCTGATTTGC-3′
	Anti-sense	5′-CCCTCTCTTCAGTATGTGACAGC-3′
**ChIP-PCR primers**		
AK015487 for Evi	Sense	5′-ACAAAGTTATATTTCACCTCCAC-3′
	Anti-sense	5′-TAGCCTCCTATCGCTTTCTAG-3′
AK016494 for Evi	Sense	5′-CAGTGGGAGGTCTGCTGTTAC-3′
	Anti-sense	5′-GCCTGCCCTACAACTACGAT-3′
AK021106 for Evi	Sense	5′-TTCCAGAGTCTCAGTGGCAGAT-3′
	Anti-sense	5′-TCGTGATCTAACATCTGAAACCC-3′
AK044545 for Evi	Sense	5′-ATAGGTGGAGCACTGAAGGC-3′
	Anti-sense	5′-ACCAACAGGAGGCAGGAGA-3′
**siRNA sequences**		
mEvi1-siRNA-1	Sense	5′-CCCGCUUGAAGCUUUGAAATT-3′
	Anti-sense	5′-UUUCAAAGCUUCAAGCGGGTT-3′
mEvi1-siRNA-2	Sense	5′-GCAACCUUCAGCGACACAUTT-3′
	Anti-sense	5′-AUGUGUCGCUGAAGGUUGCTT-3′
hEVI1-siRNA-1	Sense	5′-UCUAAGGCUGAACUAGCAGTT-3′
	Anti-sense	5′-CUGCUAGUUCAGCCUUAGATT-3′
hEVI1-siRNA-2	Sense	5′-GCUGAUUGCAGAACCCAAATT-3′
	Anti-sense	5′-UUUGGGUUCUGCAAUCAGCTT-3′
HBx-siRNA-1	Sense	5′-GCACUUCGCUUCACCUCUGTT-3′
	Anti-sense	5′-CAGAGGUGAAGCGAAGUGCTT-3′
HBx-siRNA-2	Sense	5′-CCUUGAGGCAUACUUCAAATT-3′
	Anti-sense	5′-UUUGAAGUAUGCCUCAAGGTT-3′
HBs-siRNA	Sense	5′-CCAAACCUUCGGACGGAAATT-3′
	Anti-sense	5′-UUUCCGUCCGAAGGUUUGGTT-3′
HBc-siRNA	Sense	5′-CCUUCUGACUUCUUUCCUUTT-3′
	Anti-sense	5′-AAGGAAAGAAGUCAGAAGGTT-3′

AK015487-siRNA	Sense	5′-GUCCAAAGCUCACUAUAUATT-3′
	Anti-sense	5′-UAUAUAGUGAGCUUUGGACTT-3′
Negative control	Sense	5′-UUCUCCGAACGUGUCACGUTT-3′
	Anti-sense	5′-ACGUGACACGUUCGGAGAATT-3′
**RACE gene-specific primers**		
AK015487	5'RACE	5′-CAGTTGGTAAGGAGGGGACCAAGCTTTTC-3′
	3'RACE	5′-AGGAGATGGCGAGACCCATAGCAAATGCT-3′
**probes for EMSA**		
labeled probe	Sense	5′-AAGGAAAGAATAGAATAGAACGGC-3′
	Anti-sense	5′-GCCGTTCTATTCTATTCTTTCCTT-3′
mut unlabeled probe	Sense	5′-AAGGAAATCCTCATCTAGAACGGC-3′
	Anti-sense	5′-GCCGTTCTAGATGAGGATTTCCTT-3′
unlabeled probe	Sense	5′-AAGGAAAGAATAGAATAGAACGGC-3′
	Anti-sense	5′-GCCGTTCTATTCTATTCTTTCCTT-3′
